# Socioeconomic risk markers of arthropod-borne virus (arbovirus) infections: a systematic literature review and meta-analysis

**DOI:** 10.1136/bmjgh-2021-007735

**Published:** 2022-04-14

**Authors:** Grace M Power, Aisling M Vaughan, Luxi Qiao, Nuria Sanchez Clemente, Julia M. Pescarini, Enny S. Paixão, Ludmila Lobkowicz, Amber I. Raja, André Portela Souza, Mauricio Lima Barreto, Elizabeth B. Brickley

**Affiliations:** 1 Health Equity Action Lab, Department of Infectious Disease Epidemiology, London School of Hygiene & Tropical Medicine, London, UK; 2 Department of Disease Control, London School of Hygiene & Tropical Medicine, London, UK; 3 MRC Integrative Epidemiology Unit, Population Health Sciences, Bristol Medical School, University of Bristol, Bristol, UK; 4 Washington University in St. Louis School of Medicine, St. Louis, MO, USA; 5 São Paulo School of Economics and Center for Applied Microeconomic Studies, Getulio Vargas Foundation, São Paulo, Brazil; 6 Centro de Integração de Dados e Conhecimentos para Saúde, Oswaldo Cruz Foundation, Salvador, Brazil

**Keywords:** systematic review, arboviruses, public health, epidemiology

## Abstract

**Introduction:**

Arthropod-borne viruses (arboviruses) are of notable public health importance worldwide, owing to their potential to cause explosive outbreaks and induce debilitating and potentially life-threatening disease manifestations. This systematic review and meta-analysis aims to assess the relationship between markers of socioeconomic position (SEP) and infection due to arboviruses with mosquito vectors.

**Methods:**

We conducted a systematic search on PubMed, Embase, and LILACS databases to identify studies published between 1980 and 2020 that measured the association of SEP markers with arbovirus infection. We included observational studies without geographic location or age restrictions. We excluded studies from grey literature, reviews and ecological studies. Study findings were extracted and summarised, and pooled estimates were obtained using random-effects meta-analyses.

**Results:**

We identified 36 observational studies using data pertaining to 106 524 study participants in 23 geographic locations that empirically examined the relationship between socioeconomic factors and infections caused by seven arboviruses (dengue, chikungunya, Japanese encephalitis, Rift Valley fever, Sindbis, West Nile and Zika viruses). While results were varied, descriptive synthesis pointed to a higher risk of arbovirus infection associated with markers of lower SEP, including lower education, income poverty, low healthcare coverage, poor housing materials, interrupted water supply, marital status (married, divorced or widowed), non-white ethnicities and migration status. Pooled crude estimates indicated an increased risk of arboviral infection associated with lower education (risk ratio, RR 1.5 95% CI 1.3 to 1.9); I^2^=83.1%), interruption of water supply (RR 1.2; 95% CI 1.1 to 1.3; I^2^=0.0%) and having been married (RR 1.5 95% CI 1.1 to 2.1; I^2^=85.2%).

**Conclusion:**

Evidence from this systematic review suggests that lower SEP increases the risk of acquiring arboviral infection; however, there was large heterogeneity across studies. Further studies are required to delineate the relationship between specific individual, household and community-level SEP indicators and arbovirus infection risks to help inform targeted public health interventions.

**PROSPERO registration number:**

CRD42019158572.

**Key questions**

**What is already known?**

- Arboviruses with mosquito vectors are of notable global public health importance owing to their potential to cause explosive outbreaks and induce debilitating and potentially life-threatening disease manifestations.
- In regions with established arboviral circulation, factors indicative of socioeconomic position, such as increased population density, inadequate water management and poor housing conditions, may exacerbate vector proliferation and elevate infection risks.

**What are the new findings?**

- Descriptive synthesis pointed to a higher risk of arboviral infection associated with markers of lower socioeconomic position, including lower education, income poverty, low healthcare coverage, poor housing materials, interruptions of water supply, marital status (married, divorced or widowed) and non-white ethnicity.
- Pooled crude estimates from meta-analyses indicated an increased risk of arboviral infection associated with having lower education, interruption of water supply and having ever been married.

**What do the new findings imply?**

- This review underscores the importance of evaluating the arbovirus-related impacts of social protection policies that aim to reduce the consequences of poverty (eg, conditional cash transfer, housing and public works programmes) alongside continuing research on more conventional vector control interventions.

## Introduction

Arthropod-borne viruses (arboviruses) are transmitted between vertebrate hosts by haematophagous (blood-feeding) arthropod vectors, including mosquitoes and ticks.^1^ Arboviruses with mosquito vectors, such as dengue virus (DENV) and chikungunya virus (CHIKV), are of notable public health importance worldwide owing to their potential to cause explosive outbreaks and induce debilitating and potentially life-threatening disease manifestations.^2^ In addition, congenital arboviral infections, such as with Zika virus (ZIKV), may result in severe congenital malformations with the potential to incur lifelong health and social costs for affected individuals and their families.^1–4^

Infection due to arboviruses with mosquito vectors is becoming increasingly prevalent. The burden of DENV has grown dramatically in recent decades, with substantial impact on morbidity and mortality worldwide, and ZIKV, CHIKV and Yellow Fever virus (YFV) have re-emerged.^5^ Environmental factors, such as climate change (eg, rising temperatures) and habitat modification (eg, deforestation) along with social factors, such as increased international mobility, contribute to the global spread of competent vectors and arboviruses.^6 7^ In regions with established arboviral circulation, community-level factors, such as increased population density, inadequate water management, and poor housing, may exacerbate vector proliferation and elevate infection risks.^8^ This has been reported by several ecological studies, which have shown increased levels of arboviral infections in economically deprived areas at the population-level.^9–11^ Furthermore, a recent systematic review employing descriptive synthesis reported a greater presence of *Aedes* mosquito vectors and associated arboviral diseases in regions with lower socioeconomic conditions in 50%–60% of evaluated studies.^12^ As described in the early social epidemiology literature, steep inverse associations between social class and mortality from a wide range of diseases exist.^13^ To better understand individual- and household-level risk factors for arboviral infections, we conducted a systematic review and meta-analysis synthesising published evidence on the relationship between markers of socioeconomic position (SEP) and infection due to arboviruses with mosquito vectors.

## Methods

### Search strategy and eligibility criteria

The protocol for this systematic literature review was registered in the International Prospective Register of Systematic Reviews (PROSPERO) as CRD42019158572 and was conducted in line with the 2009 Preferred Reporting Items for Systematic Reviews and Meta-Analyses (PRISMA) guidelines.^14^ We searched for studies measuring the association between SEP and arboviral infection published between 1 January 1980 and 30 June 2020 in MEDLINE (PubMed), Embase (Ovid) and LILACS (see online supplemental material 1), hypothesising that studies published more than 40 years prior to this work would lack relevance to current research. The search and full-text review were restricted to articles published in English, Portuguese, Spanish and French. Studies were eligible from any geographic location and with individuals from any age group, and included peer-reviewed observational case reports, case series or studies that had a cross-sectional, case–control or cohort study design. Studies assessing the association between SEP and/or proxy measures of SEP (eg, individual social class, living conditions, education, employment, household income, race/ethnicity and asset ownership) at the individual-level or household-level and the occurrence of acute, recent or past arboviral infection, indicated by laboratory confirmation, were included. Laboratory confirmation of arbovirus infection was based on the presence of viral RNA, antigen and/or serological evidence (eg, IgM or IgG); the quality of assays used in the individual studies was not appraised. Studies from grey literature, using an ecological design, evaluating the economic burden of arboviral infections, or only describing the natural history of disease were excluded (online supplemental material 2).

10.1136/bmjgh-2021-007735.supp1 Supplementary data

10.1136/bmjgh-2021-007735.supp2 Supplementary data

### Data extraction and meta-analysis

Data on the author, year of publication, study period, study type, source of population, data source, duration of follow-up (if applicable), geographic location, age, sex, individual-level and household-level socioeconomic characteristics, arbovirus infection type, comparison groups, confounders, frequency (number and percentage) and effect estimates (risk ratio (RR) or odds ratio (OR)) were extracted from studies and consolidated. Data screening was conducted in duplicate by four investigators (GMP, LQ, JMP and NSC) and extraction in duplicate by two investigators (GMP and AV). Discrepancies were resolved by consensus. Two reviewers (GMP and LQ) evaluated study quality by conducting a bias assessment using the Newcastle-Ottawa scale (NOS) for individual-level studies (NOS ranges from zero to nine). The NOS form for cohort studies was also used to evaluate data quality for cross-sectional studies; however, the maximum score is limited to six as it was not possible to demonstrate absence of infection at the start of these studies due to the lack of follow-up (online supplemental table 1). Evaluation was performed in duplicate, and discrepancies were resolved by consensus.

10.1136/bmjgh-2021-007735.supp3 Supplementary data

When effect estimates were provided for an indicator with comparable parameters in at least three cohort and/or cross-sectional studies, pooled effect sizes and the 95% CIs were calculated using random-effects meta-analyses. Since studies were highly heterogeneous, a random-effects model was preferred.^15^ Heterogeneity in RR estimates were assessed using I^2^ statistics and Cochran’s Q test p values. Case–control studies were not included in the meta-analyses since ORs with 95% CIs were calculated from these study data and, given the high frequency of infections in study populations, were considered to be not directly comparable with cohort and/or cross-sectional relative risk (RR) effect estimates. Further subgroup analyses were conducted for each virus within each of the meta-analyses. Analyses were performed using STATA (V.14.0). A map indicating locations where studies were based was created using Tableau software.

### Patient and public involvement

The patients and the public were not involved in the design, conduct or reporting of our research.

## Results

**Figure 1:**
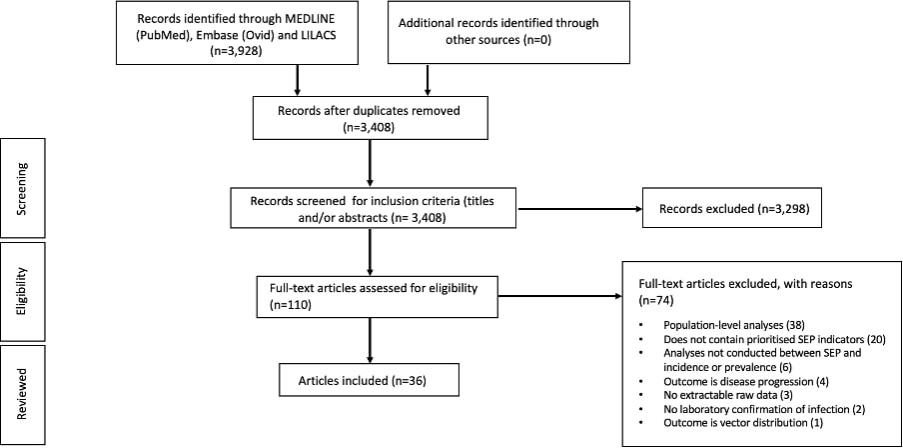
PRISMA flow chart illustrating selection of studies. PRISMA, Preferred Reporting Items for Systematic Reviews and Meta-Analyses; SEP, socioeconomic position.

Our search generated 3928 published records. After screening titles and abstracts, 110 manuscripts were assessed for eligibility. Of these, 36 articles were deemed eligible for inclusion in this systematic review (figure 1).

**Figure 2:**
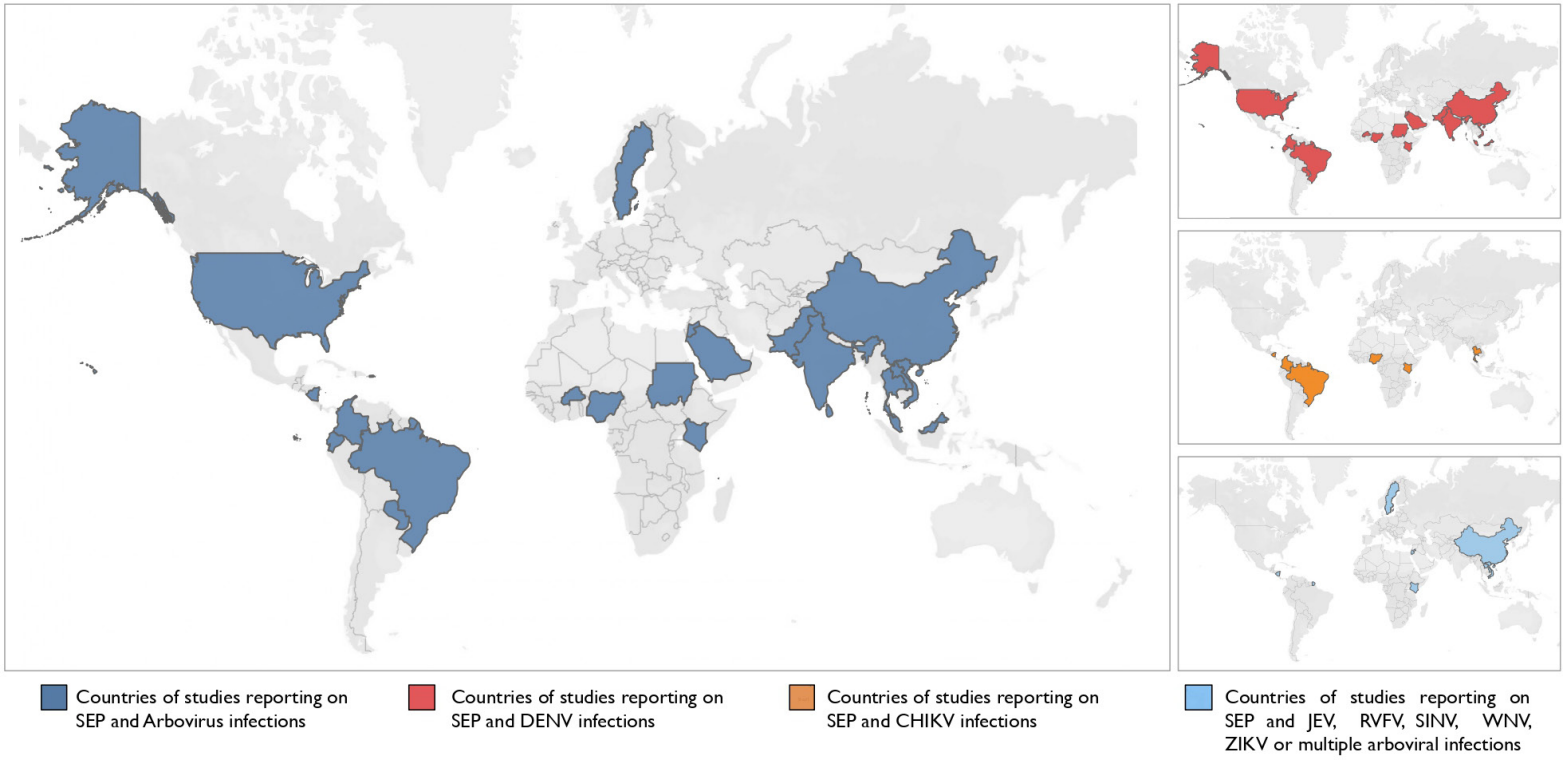
Geographic distribution of studies included in the systematic review. (A) All countries reporting SEP and arboviral infections, (B) Countries reporting SEP and Dengue virus (DENV) infections, (C) Countries reporting SEP and Chikungunya virus infections, (D) Countries reporting on SEP and Japanese encephalitis virus (JEV), Rift Valley fever virus (RVFV), Sindbis virus (SINV), West Nile virus (WNV), Zika virus (ZIKV) or multiple arboviral infections. SEP, socioeconomic position.

All studies included in this review were published between 1995 and 2020, the majority of which were published between 2015 and 2020 (n=28) and focused on DENV (n=21), CHIKV (n=6), Japanese encephalitis (JEV) (n=1), Sindbis virus (SINV) (n=1), West Nile virus (WNV) (n=1), ZIKV (n=1), DENV and JEV (n=2), DENV, CHIKV and Rift Valley fever virus (RVFV) (n=1) and flaviviruses in general with other arboviruses (n=2) (table 1, online supplemental table 2). There were no studies examining YFV. Included studies consisted of 2 cohort studies,^16 17^ 4 case–control studies,^18–21^ 27 cross-sectional studies,^22–48^ 1 nested cross-sectional study within a cohort,^49^ 1 combined cross-sectional and cohort study^50^ and 1 longitudinal serosurvey.^51^ Studies were conducted in 23 countries: 4 in low-income countries (Burkina Faso,^42^ Laos^35^ and Sudan^26 43^), 14 in lower-middle-income countries (Ecuador,^41^ India,^19^ Jordan,^33 37^ Kenya,^17 36^ Nicaragua,^16 50^ Nigeria,^27 31 40^ Pakistan,^39^ Sri Lanka^18^ and Vietnam^34^), 13 in upper-middle income (Brazil,^23 30 45–47^ China,^20 24 38^ Colombia,^49 51^ Malaysia,^25^ Paraguay^44^ and Thailand^29^) and 5 in high-income countries/territories (Mayotte (France),^28^ French Guiana,^21^ Saudi Arabia,^22^ Sweden^32^ and USA^48^) according to the Development Assistance Committee List of Official Development Assistance Recipients (figure 2).

10.1136/bmjgh-2021-007735.supp4 Supplementary data

**Table 1:** Characteristics of included studies

Author (year)	Country/ territory	Study period	Type of study	Population	Type of infection	Diagnostic test	Age range	Total size	Frequency measure	Cumulative incidence	NOS
DENV											
Brunkard *et al* (2007)^48^	USA	October 2004–November 2004	Cross-sectional	Probability-based, household selection stratified, multistage, cluster-sampling design	DENV	DENV IgM+; DENV IgG+	All ages	600	P	2%–7.3%; 40%–78%	5
da Silva-Nunes *et al* (2008)^47^	Brazil	2004–2006	Cross-sectional	Households in Ramal do Granada, were visited between March and April 2004. 466 dwellers <1–90 years of age (98.5% of the 473 areas permanent residents) were enrolled.	DENV	DENV IgG+	All ages	405	P	18.3%	6
Pessanha *et al* (2010)^46^	Brazil	June 2006–March 2007	Cross- sectional	All residents aged over 1 year in the three Belo Horizonte districts (Venda Nova, DS Leste and DS Centro-Sul)	DENV	Not specified	All ages	709	P	11.9% (95% CI 9.7% to 14.6%)	5
Kikuti *et al* (2015)^45^	Brazil	2009–2010	Cross-sectional	Individuals seeking medical care for acute febrile illness at the only public emergency health unit	DENV	DENV IgM+ and/or RT-PCR+	>5 years	2962	I	22.0%	5
Pereira *et al* (2015)^44^	Paraguay	2014	Cross-sectional	Inhabitants of three villages	DENV	DENV IgG+	All ages	418	P	24.2% (95% CI 20.2% to 28.6%)	5
Soghaier *et al* (2015)^43^	Sudan	2011	Cross-sectional	Randomly selected community population through multi-stage cluster sampling	DENV	DENV IgG+	All ages	540	P	9.4%	6
Fournet *et al* (2016)^42^	Burkina Faso	May 2004–September 2004	Cross-sectional	Children from Ouagadougou districts with different types and degrees of urbanisation	DENV	DENV IgG+	0–12 years	3015	P	22.7%	6
Kenneson *et al* (2017)^41^	Ecuador	2014–2015	Cross-sectional	Individuals with DENV infections from sentinel clinics - as well as members of the same household and four neighbouring households located within 200 meters	DENV	DENV NS1 RDT+, RT-PCR+ and/or IgM+	All ages	219	P	36.5%	5
Nasir *et al* (2017)^40^	Nigeria	May 2016–August 2016	Cross-sectional	Patients with febrile illnesses seeking medical assistance at hospital	DENV	DENV NS1 RDT+; DENV IgG+	1–49 years	171	P	8.8%; 43.3%	3
Khan *et al* (2018)^39^	Pakistan	2013–2015	Cross-sectional	DENV patient samples	DENV	DENV RT-PCR+	All ages	59 765	I	9.2%	4
Liu *et al* (2018)^38^	China	2013–2015	Cross-sectional	Samples selected from a 200,000-sample database holding serum collected from community residents living in Liwan and Yuexiu districts of Guangzhou	DENV	DENV IgM+; DENV IgG+	All ages	2085	P	3.98%; 11.8%	3
Obaidat and Roess (2018)^37^	Jordan	2015–2016	Cross-sectional	Healthy relatives of patients at governmental human health centres at 11 governorates	DENV	DENV IgG+	0–80 years	892	P	24.6%	6
Piedrahita *et al* (2018)^51^	Colombia	2010–2012	Longitudinal serosurvey	School children	DENV	DENV IgG+	5–19 years	4385	I	53.8% (2010) to 64.6% (2012)	5
Udayanga *et al* (2018)^18^	Sri Lanka	February 2017– April 2017	Case–control	Random selection of 200 households reporting past dengue incidence and 200 non-dengue reported households	DENV	N/A	All ages	4000	N/A	N/A	4
Al-Raddadi *et al* (2019)^22^	Saudi Arabia	2017	Cross-sectional	Residents of the four cities of all genders, age groups, and socioeconomic classes	DENV	DENV IgG+	All ages	6397	P	26.7%	6
Chiaravalloti-Neto *et al* (2019)^23^	Brazil	October 2015–March 2016	Cross-sectional	Residents of Vila Toninho neighbourhood	DENV	DENV IgG+	>10 y	1322	P	74.6%	8
Jing *et al* (2019)^24^	China	2015	Cross-sectional	850 participants from seven selected communities in Guangzhou with no reported dengue cases before 2014	DENV	DENV IgG+	1-84y	850	P	6.6%	6
Abd-Jamil *et al* (2020)^25^	Malaysia	2007–2010	Cross-sectional	Orange Asli populations residing in eight different villages in the forest or forest fringe areas of Peninsular Malaysia	DENV	DENV IgG+	All ages	491	P	17.0%	6
Eldigail *et al* (2020)^26^	Sudan	August 2017–May 2018	Cross-sectional	Eleven localities of Kassala state	DENV	DENV IgG+	All ages	600	P	11.4%	6
Omatola *et al* (2020)^31^	Nigeria	2019	Cross-sectional	Visiting outpatients from the four hospitals in Anyigba	DENV	DENV IgG+	All ages	200	P	20.5%	3
Swain *et al* (2020)^19^	India	2017	Case–control	Confirmed dengue patients within 1 year in six districts of the state	DENV	DENV IgM+	All ages	767	N/A	N/A	8
CHIKV											
Sissoko *et al* (2008)^28^	Mayotte	2005–2006	Cross-sectional	Household-based; complex multistage cluster sampling of population of Mayotte	CHIKV	CHIKV IgG+	≥2 years	1154	P	37.2%	6
Nakkhara *et al* (2013)^29^	Thailand	2008	Cross-sectional	Residents aged 18 years or more from three villages	CHIKV	CHIKV IgG+	>18 years	507	P	61.9%	5
Kuan *et al* (2016)^50^	Nicaragua	March 2015–April 2016	Cross-sectional; Cohort	Children aged 2–14 years enrolled in the Paediatric Dengue Cohort Study; Household recruitment	CHIKV	CHIKV total antibody+	2–14 years; >15 years	3362; 848	P	6.1% (2-14 years); 13.1% (>15 years)	9; 5
Rueda *et al* (2019)^49^	Colombia	2014	Cross-sectional nested in community cohort	548 suspected CHIKV patients from the COPCORD cohort	CHIKV	CHIKV IgG+	>18 years	548	P	53.8%	4
Anjos *et al* (2020)^30^	Brazil	2016–2017	Cross-sectional	All households of 3 contiguous valleys in Pau da Lima who are ≥5 years of age	CHIKV	CHIKV IgM+, CHIKV IgG+	All ages	1772	P	11.8%	4
Omatola *et al* (2020)^27^	Nigeria	2018	Cross-sectional	Febrile participants at five hospitals in Anyigba who test negative for typhoid and malaria	CHIKV	CHIKV IgM+, CHIKV IgG+	All ages	243	P	34.2%	3
JEV											
Luo *et al* (1995)^20^	China	June 1991–September 1991	Case–control	Active case finding in hospitals in Gusi County, Henan, China	JEV	JEV IgG+	>6 months - 10 years	150	N/A	N/A	8
SINV											
Ahlm *et al* (2014)^32^	Sweden	2009	Cross-sectional	Randomly selected from population registers	SINV	SINV IgG+	25–75 years	1729	P	2.9%	6
WNV											
Obaidat *et al* (2019)^33^	Jordan	November 2015–May 2016	Cross-sectional	Healthy relatives of patients seeking healthcare at health centres throughout Jordan.	WNV	WNV IgG+	15–50 years	801	P	8.6%	6
ZIKV											
Burger-Calderon *et al* (2018)^16^	Nicaragua	August 2016–October 2016	Cohort	Laboratory-confirmed Zika index cases and their household members	ZIKV	ZIKV RT-PCR+	All ages	142	I	31.0%	8
Multiple arboviruses											
Bartley *et al* (2002)^34^	Viet Nam	April 1996–August 1997	Cross-sectional	Community and hospital-based subjects	DENV; JEV	DENV or JEV IgG+	All ages	308	P	66.0%	5
Conlan *et al* (2015)^35^	Laos	January 2009–March 2009	Cross-sectional	Random selection of 14 households per village and all household members over 6 years age asked to participate	JEV; DENV	NC; JEV HI+; DENV1 HI+; DENV2 HI+; DENV3 HI+; DENV4 HI+	≥6 years	1136	P	67.3% (Any flavivirus); 39.4% (JEV); 2.2% (DENV 1); 0.8% (DENV2); 0.8% (DENV3); 13.6% (DENV4)	4
Ochieng *et al* (2015)^36^	Kenya	2007	Cross-sectional	HIV-negative blood specimens from the 2007 Kenya AIDS Indicator Survey	CHIKV; DENV; RVFV	CHIKV IgG+; DENV IgG+; RVFV IgG+	15–64 years	1091	P	0.97%; 12.5%; 4.5%	3
Bonifay *et al* (2017)^21^	French Guiana	March 2013–June 2014	Case–controlE	Group of patients infected with CHIKV in 2014 with a group infected with DENV	CHIKV; DENV	CHIKV RT-PCR+; DENV IgM+	>15 years and 3 months	336	N/A	N/A	6
Hortion *et al* (2019)^17^	Kenya	December 2014–December 2015	Cohort	Acutely ill children presenting at one of four healthcare centres	Flavivirus, CHIKV; DENV	CHIKV IgG+; DENV IgG+	All ages	1604	P	3.7%	6

*The authors report it was not possible to distinguish between DENV and JEV IgG due to cross-reactivity.

CHIKV, Chikungunya virus; DENV, Dengue virus; HI, Hemagglutination inhibition; I, Incidence; Ig, Immunoglobulin; JEV, Japanese Encephalitis virus; N/A, not applicable; NC, not clear; NOS, Newcastle-Ottawa scale; NS1, Non-structural protein 1; P, Prevalence; RDT, Rapid diagnostic test; SINV, Sindbis virus; WNV, West Nile virus; ZIKV, Zika virus.

### Age and sex

Age and sex were investigated and/or adjusted for in 32 of the 36 studies on seven arboviruses (CHIKV, DENV, JEV, RVFV, SINV, WNV and ZIKV). These studies included three case–control, two cohort, 25 cross-sectional studies, one study comprising a cross-sectional and cohort investigation^50^ and 1 cross-sectional nested in a cohort study, spanning 21 countries.

Of the 20 studies that evaluated the relationship between age and arboviral infection, 18 (90%) reported evidence of an association between increasing age and seropositivity for arboviruses, while four studies (20%) found statistical evidence of an association between age and past arboviral infection (DENV^23 36 37^ and CHIKV^50^) in adjusted models.

**Figure 3:**
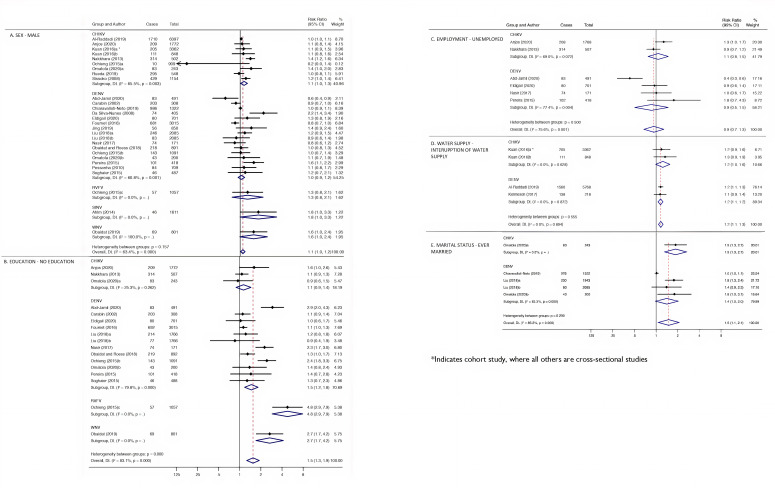
Meta-analysis for the association between socioeconomic risk markers and arboviral infections. Pooled estimates using random-effects meta-analyses are calculated by subgroups of socioeconomic markers, sex (A), education (B), employment (C), water supply (D) and marital status (E). Subgroups of arboviruses are additionally presented per risk marker. Error bars show the point RR with their 95% CIs on the log scale for each study. Diamonds show the combined point estimate. I^2^ statistics and Q-test p values are reported. *Indicates cohort study, whereas all others are cross-sectional studies.

All 36 studies considered the direct relationship between sex and arboviral infection or adjusted for sex in the model. Five (13.9%) of these studies reported evidence of higher prevalence of arboviruses among males in crude analyses.^28 32 39 45 47^ However, statistical analyses were not provided for every study, and just eight provided an adjusted point estimate.^16 23 34 36 37 47 50 51^ A study conducted in Sweden^32^ found a crude statistical association between being male and seropositivity for SINV; however, on adjusting for age and smoking in multivariate analyses, neither sex nor age were significant predictors of seropositivity for SINV. Twenty-four studies with 28 crude estimates comprising a total of 34 373 individuals were included in the random-effects meta-analysis of the association of sex and arboviral infection. The crude combined RR for males was 1.1 (95% CI 1.0 to 1.2), with substantial heterogeneity between studies (I^2^=63.4%) (figure 3A). Disease-specific pooled estimates indicated a RR of 1.1 (95% CI 1.0 to 1.3) and 1.0 (95% CI 0.9 to 1.2) in CHIKV and DENV subgroups, respectively.

### Education and occupation

The association between education and arboviral infection was analysed in 1 cross-sectional study nested in a cohort, 2 case–control and 22 cross-sectional studies, spanning 18 countries and 6 arboviruses (CHIKV, DENV, JEV, RVFV, SINV and WNV). In these studies, education was classified in distinct ways depending on context, and included level of education,^19 24 26 27 29 31–34 36 38–41 43 44 49^ schooling age,^23^ parental education,^20^ the attainment of any formal education,^25 37 42^ length of education in years^28^ and illiteracy.^30 45^

Overall, there tended to be a higher risk of infection among less educated individuals in crude analyses. However, studies that developed multivariate models indicated weak or no statistical evidence of an association between education and arboviral infection after accounting for confounding factors.^19 20 23 32 36 37^ In addition, a cross-sectional study conducted in China presented evidence that fewer years of parental schooling was associated with increased risk of JEV infection;^20^ however, on adjusting for JEV vaccination, there was very little evidence remaining. In the 17 investigations (n=15 760) included in the random-effects meta-analysis for education, the crude combined RR for lack of education was 1.5 (95% CI 1.3 to 1.9); however, there was considerable heterogeneity between studies (I^2^=83.1%) (figure 3B).

Random-effects meta-analysis for disease-specific pooled estimates revealed that individuals with no education had a crude combined RR of 1.5 (95% CI 1.2 to 1.8) for DENV infections and 1.1 (95% CI 0.9 to 1.4) for CHIKV infections.

Occupation was assessed in 11 cross-sectional studies and 1 case–control study. Eleven of the 12 studies presented frequencies, 6 presented crude effect estimates and 2 presented adjusted effect estimates. The occupation-related variables analysed were employment status,^25 26 30^ location of work (inside or outside),^23^ earnings (above the country’s minimum wage or not),^41^ employment stability and occupation types.^19 27–29 31 40 44^ In a study conducted by Chiaravalloti-Neto *et al* in Brazil, there was a crude association between working outside and seropositivity for DENV, which was lost on adjusting for other socioeconomic and demographic covariates.^23^ Swain *et al* indicated evidence to suggest that DENV infection was associated with occupations that required travel into certain parts of India.^19^ Collectively, in the six studies (n=4056) that were included in the random-effects meta-analysis for occupation, there was little evidence of an association between lack of employment and arboviral infection (pooled RR 0.9; CI 95% 0.7 to 1.3), with considerable heterogeneity between studies (I^2^=75.6%) (figure 3C).

### Income poverty and social vulnerability

Variables indicating income poverty and social vulnerability varied considerably and thus were challenging to standardise; however, descriptive analyses indicate that lower income was a risk factor for arboviral infection, with limited empirical evidence.

The relationship between poverty or social vulnerability and arboviral infection was assessed in 1 cohort, 4 case–controls and 15 cross-sectional studies, across 16 countries and 4 arboviruses (CHIKV, DENV, JEV and WNV). Assessments were based on weekly or monthly household income,^18 20 23 25 26 33 39 44–46 48 49^ SEP categorised into groups,^42 49 50^ per capita income quartiles or quintiles.^35 36 47^ Health vulnerability was also assessed in two studies.^21 46^ This comprised estimating a health vulnerability index and health vulnerability through state or free care compared with social security and complimentary health insurance. Frequencies and/or effect estimates were extracted for 14. Four studies investigating DENV found evidence of a relationship between lower household income and increased arboviral infection.^25 45 47 48^ One case–control study, conducted in French Guiana, that specifically examined healthcare coverage status in relation to CHIKV and DENV infection, found that a lack of private health insurance was associated with higher CHIKV infection both in the crude and adjusted analyses. In contrast, however, DENV appears to affect a wealthier population.^21^ Since poverty indicators were not measured consistently between studies and study contexts, a meta-analysis was not possible for income or social vulnerability factors in this study.

### Household conditions

Four case–control, three cohort, one longitudinal serosurvey and 18 cross-sectional studies investigated the association between household characteristics and arboviral infections. These studies examined the type or size of residence,^19 22–24 30 32 34 44 46^ house appearance or quality,^20 28 42^ number of rooms,^22 41^ building density,^42^ household crowding,^17 18 22 23 28 30 31 41 43 44 48 50^ type or presence of walls,^47^ wall gaps,^47^ presence of screens,^41 48^ residential area,^17 21 32 37^ waste management^42 45^ and asset ownership (air conditioning,^48^ refrigerator,^16^ television,^34^ land tenure and home ownership^23 41 47^ and asset ownership index (presence of electricity, flush toilet, piped water and possession of a television set, radio or refrigerator).^28^

Of the four studies that evaluated the association between type of residential area (urban vs rural) and arboviral infections,^17 32 34 37^ one reported higher risk of SINV infection in small, rural residential areas in Northern Sweden,^32^ one study showed that the risk of flavivirus infection was higher in urban residential areas or cities compared with surrounding rural areas and Southern Vietnam,^34^ while a study in Kenya observed no difference in flavivirus infection between rural and urban areas but did note a higher seroprevalence among coastal compared with western study participants.^17^ In Jordan, a higher risk of WNV infection was reported for those living in Badia and the Jordan Valley regions (arid and hot climates) compared with those living in the Highlands and Plains regions (colder and higher precipitation areas).^37^

The relationship between house or land ownership and arboviral infection was evaluated in three studies.^23 41 47^ A cross-sectional study conducted in Brazil showed little evidence of an association between home ownership and seropositivity in DENV, although living in a house compared with an apartment was positively associated with DENV seropositivity, after adjusting for socioeconomic and demographic covariates.^23^ Crude analyses indicated evidence of a negative association between land tenure in rural Amazonia, Brazil, and DENV seropositivity; however, this association was weak in the adjusted analysis.^47^

Of the seven studies that analysed building materials, three studies found an association between poor building materials or structures and arboviral infection.^20 28 30^ In addition, unstructured low building density households had higher prevalences of CHIKV and DENV.^19 20 28 42^

Crowding, categorised by number of individuals per household,^17 22 23 28 30 43 44 48 50^ residents per room^41^ or residents per bed^27^ was analysed in 11 studies, of which four found an association between crowding and arboviral infection.^23 28 43 50^ In a study conducted in Paraguay, DENV prevalence was higher for those who lived alone compared with those who lived with others.^44^

### Water supply and sanitation

Water supply or service consumption was investigated in eight studies^16 22 37 41–44 50^ and waste collection or sanitation in three studies.^22 42 48^ Having adequate water supply (ie, tap or piped water) was associated with lower DENV infection in Ecuador^41^ and Paraguay.^44^ In addition, water supplied by water wells, onsite water storage and frequent/longer interruptions of water supply was associated with higher flavivirus seroprevalence in Burkina Faso,^42^ higher seropositivity for ZIKV in contacts of ZIKV index cases in Nicaragua,^16^ higher DENV infection in Ecuador^41^ and Saudi Arabia,^22^ and higher CHIKV infection in children in Nicaragua.^50^

Improper waste management practices were also significantly associated with flavivirus IgG in different building density strata in Burkina Faso,^42^ while an association was found between lack of street drainage and higher DENV infection on the US/Mexico border.^48^ The absence of sanitation was strongly associated with DENV infection in crude analysis in Saudi Arabia; however, this was not included in the multivariable analysis.^22^ The random-effects meta-analysis from three studies (one of which contained a cohort (A) and cross-sectional (B) study design) (n=10 196) revealed evidence of an association between interruption of water supply and arboviral infection (RR 1.2; 95% CI 1.1 to 1.3; I^2^=0.0%) (figure 3D).

### Other (marital status, ethnicity and migration status)

A range of other sociodemographic factors that act as proxies for SEP were investigated by several articles identified in this review. Having been born overseas was associated with greater risk of past arboviral infection, evidenced by one study,^21^ and crude analyses indicated individuals who identified as non-white or of a schedule caste in India, had a higher risk of arboviral infection.^19 23 45 49^ The evidence was limited, concentrated in six countries and largely focused on DENV or CHIKV.

Having been married, including currently or previously (ie, divorced or widowed), was associated with an overall increase in risk of arbovirus infection.^23 31 38^ Marital status and its association with DENV and CHIKV IgG and/or IgM antibody levels was investigated in four cross-sectional studies, conducted in Guangzhou, China,^38^ São Paulo, Brazil,^23^ Guinea Savannah, Nigeria,^31^ and Kogi state, Nigeria.^27^ In São Paulo,^23^ adjusted analyses showed that being single was a risk factor for DENV compared with being married, while in Guangzhou, China,^38^ crude analyses showed that widowed or divorced individuals were at higher risk of infection compared with both their married and single counterparts. Adjusted analyses from these two studies, however, revealed no statistical evidence of an association. All four studies were included in the random-effects meta-analysis, which revealed statistical evidence that individuals who had ever been married, including currently married, divorced or widowed, had higher overall crude risks of arboviral infection (RR 1.5 95% CI 1.1 to 2.1; I^2^=85.2%) than those who were single (figure 3E).

Four studies examined race/caste as a correlate of arboviral infection, of which two were conducted in Brazil,^23 45^ one in Colombia^49^ and one in India.^19^ The two Brazilian studies found that Black and non-white individuals were at increased risk of DENV^23 45^ and a case–control study conducted in Odisha, India, revealed higher odds of DENV infection in those considered a schedule caste or schedule tribe (official term given in India to those who have historically faced deprivation, oppression and marginalisation) compared with those considered non-schedule caste or non-schedule tribe.^19^ The crude analyses showed evidence of this association; however, this was lost on adjusting for unmentioned confounders. A meta-analysis was not performed due to the heterogeneity of study contexts and the countries’ specific social constructions of race/caste.

Migration status, defined on the basis of the country of birth: French-born and Foreign-born, was investigated as a potential risk factor for arboviral infection in a case–control study conducted in French Guiana.^21^ This study found strong statistical evidence in crude analysis that individuals born abroad had over four times the odds of testing positive for DENV IgG than those born in French West Indies, French Guiana or Mainland France. One study additionally indicated that changing city within Brazil was not associated with an increase in DENV infection risk.^46^

### Quality evaluation

The quality scores of the 36 individual studies varied across study designs. For cross-sectional studies, scores ranged from 3 to 6, with weaknesses related to selection bias of exposed cohorts and lack of adjustment for confounders. For the cohort studies, scores ranged from 6 to 9, with weaknesses related to no indication of absence of disease at the start of the study and to lack of adjustment for confounders (online supplemental table 1A). For case–control studies, scores ranged from 4 to 8, with weaknesses related to lack of adjustment for confounders (online supplemental table 1B).

## Discussion

In this systematic review and meta-analysis, we summarised published evidence linking markers of SEP and infection due to arboviruses with mosquito vectors. Descriptive results indicated lower education, income poverty, low healthcare coverage, poor housing materials, interrupted water supply, marital status (married, single, divorced or widowed), non-white ethnicities and migration status as potential risk factors for arboviral infection. Meta-analyses provided statistical evidence of an increased risk of infection due to arboviruses with mosquito vectors associated with lack of education, interruption of water and having ever been married.

Overall, the seroprevalence of arboviral-specific antibodies (in particular, to DENV) was shown to be highest in older age groups. This finding corroborates a number of studies that found a positive association between age and seropositivity for DENV and is assumed to be related to the longer period of exposure to DENV over time.^52–58^ No clear association between arboviral infection and sex was observed.

In addition, individuals with lower education were at greater risk of arboviral infection in both the descriptive summary and meta-analysis. Education is commonly used as a generic indicator for SEP, highlighting the accumulation of advantage and disadvantage over the lifecourse.^59 60^ It is associated with permanent income status, whereas income itself, for example, captures the level of income at the time of data collection and is thus, in general, volatile. These findings, therefore, might suggest that structural poverty is a relatively more important factor than transient poverty. Education is also argued to capture the knowledge and skill-related assets of an individual, which may contribute to the receptivity of health messaging and thus permitting more informed use of vector control activities to reduce risk of infection.^61^

The descriptive analysis for employment assessed several occupations and occupational exposure types, while the meta-analysis looked at unemployment compared with being employed. No overall statistical evidence for unemployment as a risk factor for arboviral infection was apparent. The unobserved effect is likely because the degree of vulnerability linked to unemployment is highly dependent on both the type of employment (indoor or outdoor occupations) as well as the country’s overall economic circumstances.^59^ Thus, this indicator is limited when comparing across studies as well as geographic areas.

Poverty has long been considered a determinant of arboviral infections such as DENV and CHIKV; however, the scarcity of studies with consistent measures of income poverty and social vulnerability has meant that such a relationship has yet to be substantiated. Indeed, in this systematic review, a meta-analysis was not possible for the variables that indicated income poverty and social vulnerability, since contexts within which the data were collected for these were not standardised. Descriptive analyses, nonetheless, indicated that lower income appeared to be a risk factor, although with limited empirical evidence. This is additionally supported by the vast literature on social determinants of health.^62^ Income can influence a variety of material circumstances with direct implications for health and arbovirus exposure.^63^ The conversion of money and assets into health-enhancing commodities or behaviours may be more relevant to understanding how this variable affects arboviral infection directly.^59^

While a meta-analysis was not completed for the variables related to the constructs of race or caste, the descriptive analysis revealed that individuals who identified as non-white^23 45^ or of a schedule caste^19^ were at greater risk of arboviral infection. While there is no biological basis for an association between these constructs and health,^64^ ethnicity, caste and race are proxies for the embodiment of xenophobia, casteism and racism in their structural, cultural and interpersonal forms.^65^ Data from the US context, for example, observed that in areas where mortality rates are highest, the fraction of black residents is larger.^66^ These findings may be extrapolated to the Brazilian context, where racial inequality and segregation are reflected in social disadvantage^65^ and health inequities.

Substandard housing conditions are likely to lead to greater exposure to mosquitoes and thus increased risk of infection.^67^ The association between poor quality housing conditions and arboviral infection was a common finding in many of the studies assessed. However, due to the diversity of indicators relating to household conditions, it was not possible to evaluate this in a meta-analysis. Poor living conditions are often also characterised by overcrowding. Indeed, household crowding appeared to be an additional risk factor for DENV infection. While the reasons behind this are unknown, it is likely due to the association between household crowing and income poverty as well as to the higher concentration of carbon dioxide and other chemicals in crowded houses which attracts a greater number of mosquitoes.^68^ Furthermore, the meta-analysis conducted on water supply in this study provided evidence that interruption in water supply, likely resulting in storage of water in containers and creation of prime breeding spots for mosquitoes,^69^ may increase risk of CHIKV and DENV infection.

The meta-analysis provided evidence that having been married, including currently or previously (ie, divorced or widowed), was associated with an increase in arboviral infection risk; however, the descriptive analysis indicated that most of these associations diminish after adjusting for confounding. Age may be a particularly important confounder in this context. Migration was assessed in one study and presented descriptively in this analysis. Those classified as migrants were considered to be in a precarious social situation, since they did not have regular social security and health insurance and therefore were more at risk of arboviral infection.^21^

This review has strengths and limitations. First, it is among the first to conduct a systematic review and meta-analysis using diverse populations to assess SEP indicators that identify individuals at the highest risk of arboviral infection. Further research is required to understand the specific mechanisms by which these factors impact infection. The findings of this review should be interpreted with caution, since there were high levels of heterogeneity between studies, which is likely a result of differences in study design, study population and contexts within which these data were collected as well as differences inherent to the individual arboviruses and their mosquito vectors. While this review addressed several arboviruses that circulate in different ecological cycles and involve differences in vector-host preferences, local host abundances and herd immunity, assessing the social determinants of these arboviruses together allows for the analysis of distal risk factors, such as socioeconomic indicators, that have an overarching effect on all arboviral infections.^7^ However, we acknowledge that grouping findings from multiple arboviruses may obscure observations and the heterogeneity of the measures used to capture the range of socioeconomic factors analysed in these studies make it more difficult to delineate associations of interest. Furthermore, this review did not differentiate past infections from current infections and therefore changes in SEP, civil status and even location may have introduced misclassification bias.

## Conclusion

Evidence from this systematic review suggests that indicators of lower SEP at the individual and household-levels are associated with increased risks of acquiring arboviral infection across a wide range of geographic and cultural contexts. Although not a sufficient determinant of arbovirus risk in itself, poverty is closely correlated with the risk factors for arbovirus infection identified in this review. Within settings experiencing a high burden of arbovirus infections, further work is required to delineate the roles of specific socioeconomic risk factors to inform locally relevant preventive activities. More broadly, the findings of this review underscore the importance of evaluating the arbovirus-related impacts of social protection policies that aim to reduce the consequences of poverty (eg, conditional cash transfer, housing and public works programmes) alongside continuing research on more conventional vector control interventions. To conclude, the findings of this review add to relatively sparse data on the socioeconomic determinants of infection due to arboviruses with mosquito vectors and emphasise the need for further research to disrupt the cycle of poverty, vulnerability and arbovirus-related illness.

10.1136/bmjgh-2021-007735.supp5 Abstract translation This web only file has been produced by the BMJ Publishing Group from an electronic file supplied by the author(s) and has not been edited for content.

10.1136/bmjgh-2021-007735.supp6 Abstract translation This web only file has been produced by the BMJ Publishing Group from an electronic file supplied by the author(s) and has not been edited for content.

## Data Availability

All data relevant to the study are included in the article or uploaded as online supplemental information.
