# Supplementary material for: Socioeconomic risk markers of arthropod-borne virus (arbovirus) infections: a systematic literature review and meta-analysis
Source: BMJ Glob Health. 2022 Apr 14;7(4):e007735. doi: 10.1136/bmjgh-2021-007735 (PMC9014035; doi:10.1136/bmjgh-2021-007735)
Supplement: Supplementary data [file bmjgh-2021-007735supp001.pdf]

Supplementary Material 1. Search strategy used to study socioeconomic factors associated with arboviruses

Pubmed

("Arboviruses"[Mesh] OR "Arbovirus Infections"[Mesh] OR "Zika Virus"[Mesh] OR "Zika Virus Infection"[Mesh] OR "Dengue"[Mesh] OR "Severe Dengue"[Mesh] OR "Dengue Virus"[Mesh] OR "Chikungunya Fever"[Mesh] OR "Chikungunya virus"[Mesh] OR "Encephalitis, Japanese"[Mesh] OR "Encephalitis Viruses, Japanese"[Mesh] OR "Encephalitis Virus, Japanese"[Mesh] OR "Rift Valley Fever"[Mesh] OR "Rift Valley fever virus"[Mesh] OR "West Nile virus"[Mesh] OR "West Nile Fever"[Mesh] OR "Yellow Fever"[Mesh] OR "Yellow fever virus"[Mesh] OR zika[Title/Abstract] OR zikv[Title/Abstract] OR denv[Title/Abstract] OR dengue[Title/Abstract] OR chikv[Title/Abstract] OR chikungunya[Title/Abstract])

AND

("Social Conditions"[Mesh] OR "Socioeconomic Factors"[Mesh] OR "Social Class"[Mesh] OR "Poverty"[Mesh] OR "Poverty Areas"[Mesh] OR "Income"[Mesh] OR "Education"[Mesh] OR "Educational Status"[Mesh] OR "Ethnic Groups"[Mesh] OR "Race Factors"[Mesh] OR socioeconomic*)

Embase

exp Arbovirus/ OR (Arboviruses or Arbovirus Infections or Zika Virus or Severe Dengue or Dengue Virus or Chikungunya Fever or Chikungunya virus or Japanese Encephalitis Viruse or Rift Valley Fever or West Nile virus or West Nile Fever or Yellow Fever or zikv or denv or chikv).mp. [mp=title, abstract, heading word, drug trade name, original title, device manufacturer, drug manufacturer, device trade name, keyword, floating subheading word, candidate term word]

AND

exp social class/ OR (Social Conditions or socioeconomic* or Social Class or Poverty or Poverty Areas or Income or Education or Educational Status or ethnic* or race).mp. [mp=title, abstract, heading word, drug trade name, original title, device manufacturer, drug manufacturer, device trade name, keyword, floating subheading word, candidate term word]

LILACS

Arboviruses or Arbovirus Infections or Zika Virus or Severe Dengue or Dengue Virus or Chikungunya Fever or Chikungunya virus or Japanese Encephalitis Viruse or Rift Valley Fever or West Nile virus or West Nile Fever or Yellow Fever or zikv or denv or chikv [Palavras]

AND

Social Conditions or Socioeconomic$ or Social Class or Poverty or Poverty Areas or Income or Education or Educational Status or ethnic$ or race or socioeconomic or pobreza or social or renda or educa$ or raca [Palavras]
