# Supplementary material for: Socioeconomic risk markers of arthropod-borne virus (arbovirus) infections: a systematic literature review and meta-analysis
Source: BMJ Glob Health. 2022 Apr 14;7(4):e007735. doi: 10.1136/bmjgh-2021-007735 (PMC9014035; doi:10.1136/bmjgh-2021-007735)
Supplement: Supplementary data [file bmjgh-2021-007735supp002.pdf]

Supplementary Material 2: Inclusion and exclusion criteria

**Inclusion Criteria**	
Types of studies	Publication date: 1980 to 2020
	Studies from any geographical location.
	English, Spanish, Portuguese and French language.
	Studies using quantitative methods: Observational case reports, case series, cross-sectional, case-control and cohort studies.
Types of participants	All ages acceptable.
Types of exposure measures	Socioeconomic position and/or proxy measures of socioeconomic position at an individual level, such as social class, living conditions, education, household income, ethnicity if directly linked to socioeconomic status and asset ownership.
Types of outcome measures	Occurrence of infections due to arboviruses with mosquito vectors

**Exclusion Criteria**	
Types of studies	Grey literature / not published in a peer reviewed journal, ecological study designs/spatial analyses with no indication of individual risk factors
	Treatment guidelines documents, other systematic reviews
Dates of studies	<1980s
Types of outcome measures	Economic burden of arbovirus (e.g., economic evaluation of costs of disease to families or governments).
