# Supplementary material for: Socioeconomic risk markers of arthropod-borne virus (arbovirus) infections: a systematic literature review and meta-analysis
Source: BMJ Glob Health. 2022 Apr 14;7(4):e007735. doi: 10.1136/bmjgh-2021-007735 (PMC9014035; doi:10.1136/bmjgh-2021-007735)
Supplement: Supplementary data [file bmjgh-2021-007735supp003.pdf]

Supplementary Table 1. Bias assessment using the Newcastle-Ottawa scale (NOS) for individual-level studies for cross-sectional and cohort (A) and case-control (B)

Author(s)	Year of	Selection (4)				Comparability (2)		Outcome (3)			Total Score	Study design	Study description
		Representativeness of the Exposed Cohort	Selection of the Non-Exposed Cohort	Ascertainment of Exposure	Demonstration That Outcomes of Interest was Not Present at Start	Comparable in Main Factor (age)	Comparability in other controlled factors (i.e. sex)	Assessment of Outcome	Sufficient period of follow up	Adequacy of follow up			
Abd-Jamil, J., et al.	2020	1	1	1		1	1	1			6	Cross sectional	This present study was performed to investigate the seroprevalence of dengue among the Orang Asli (OA) residing at the forest fringe areas of Peninsular Malaysia and determine the factors that could affect the transmission of dengue among the OA
Ahlm, C., et al.	2014	1	1	1		1	1	1			6	Cross-sectional	Seroprevalence study to determine seroprevalence of SINV and associated risk factors.
Al-Raddadi et al	2019	1	1	1		1	1	1			6	Cross sectional	To estimate the seroprevalence of dengue in these regions and the risk factors associated with positive serology
Anjos et al	2020	1	1	1		0	0	1			4	Cross sectional	Investigate factors associated with prior CHIKV infection
Bartley, L.M., et al.	2002	0 (only typhoid patients)	1	1		1	1	1			5	Cross-sectional	Study assesses associations between sociodemograpgic factors and dengue and Japanese encflavivirus seroprevalence in Southern Vietnam
Brunkard, J. M., et al.	2007	0 (more females than male)	1	1		1	1	1			5	Cross-sectional	Cross-sectional serosurvey used to assess dengue seroprevalenceon the southern Texas-Mexico border and assess assocaited risk factors
Burger-Calderon, R., et al.	2018	1	1	1	0	1	1	1	1	1	8	Cohort	Study assessing the prevalence of ZIKV and its social determinants in Nicaragua.
Chiaravalloti-Neto, F., et al.	2019	1	1	1	0	1	1	1	1	1	8	Cross-sectional/cohort	Seroprevalence study to determine seroprevalence and incidence of DENV and identify if SES and demographic covariates are associated with seropositivity
Conlan, J. V., et al.	2015	1	1	1		0	0	1			4	Cross-sectional	Seroprevalence study to determine seroprevalence of flaviviruses (JEV and DENV) and associated risk factors.
Da Silva-Nunes, M., et al.	2008	1	1	1		1	1	1			6	Cross-sectional	Seroprevalence study to determine seroporevalence of DENV in Amazonas region of Brazil and associated risk factors
Eldigail, M. H., et al	2020	1	1	1		1	1	1			6	Cross sectional	In the present investigation, a cross sectional study was conducted to advance an understanding of the prevalence of DENV and associated risk factors were determined in Kassala State, Sudan
Fournet, F., et al.	2016	1	1	1		1	1	1			6	Cross-sectional	Seroprevalence study to analyse flavivirus prevalence relative to the socioeconomic, demographic, health and environmental data concerning children, their family and household and the district
Hortion, J., et al.	2019	0 (only acutely ill patients in hospital)	1	1	1	0	0	1	1	1	6	Cohort study	This seroprevalence study aimed to investigate the frequency of alphavirus and flavivirus incident infections in two regions in Kenya and identify potential risk factors
Jing, Q., et al.	2020	1	1	1		1	1	1			6	Cross sectional	A cross-sectional serosurvey using a stratified random sampling method among individuals aged 1–84 years-old in 7 communities in Guangzhou with no reported dengue cases before 2014 was performed.
Kenneson, A., et al.	2017	0 (1/3 of cases are referred from MOH health facilities)	1	1		1	1	1			5	Cross-sectional	The authors conducted a household-level study to identify KAP and social-ecological risk factors associated with acute or recent DENV infections in the city of Machala, Ecuador
Khan, J., et al.	2018	1	1	1		0	0	1			4	Cross sectional	
Kikuti, M., et al.	2015	0 (only acutely febrile patients)	1	1		1	1	1			5	Cross-sectional	Conducted enhanced, community-based surveillance in the only public emergency unit in a slum in Salvador, Brazil to identify acute febrile illness (AFI) patients with laboratory evidence of dengue infection
Kuan, G., et al.	2016	1	1	1	1	1	1	1	1	1	9	Community based cohort (0-14)	Two studies were conducted to analyse the seroprevalence of CHIKV after the first chikungunya epidemic in a community-based cohort of children ages 2-14 years and a cross-sectional survey of persons over 15 years old in the same are of Managua, Nicaragua.
		0 (more females)	1	1		1	1	1			5	Cross-sectional (>15 yo)	
Liu, J., et al.	2018	0 (more females)	1	1		0	0	1			3	Cross-sectional	This cross-sectional study explored the sero-prevalence of dengue virus infection in Guangzhou
Nakkhara, P., et al.	2013	0 (more females)	1	1		1	1	1			5	Cross-sectional	
Nasir, I. A., et al.	2017	0 (patients with febrile illness)	1	1		0	0	1			3	Cross-sectional	
Obaidat, M. M. and A. A. Roess	2018	1	1	1		1	1	1			6	Cross-sectional	Seroprevalence study to understand the prevalence of DENV in Jordan and ssess risk factors that may be assoicated with increased seropositivity
Obaidat, M. M., et al.	2019	1	1	1		1	1	1			6	Cross-sectional	
Ochieng, C., et al.	2015	0 (only HIV negative samples)	1	1		1	0 (did not adjust for sex)	1			3	Cross-sectional	Seroprevalence study to understand the prevalence of DENV, CHIKC and RVFC in Kenya and associated risk factors
Omatola, C. A., et al.	2020	0 (Only included patients who had fever and suspected typhoid or malaria)	1	1		0	0	1			3	Cross-sectional	This study identifies past exposure to DENV among people in Anyigba, located in the Guinea Savannah region, Nigeria.
Omatola, C. A., et al.	2020	0 (Only patients with febrile illness were included)	1	1		0	0	1			3	Cross-sectional	This study identifies recent CHIKV infection in Anyigba, Nigeria.
Pereira, Y., et al	2015	1	1	1		1	0	1			5	Cross-sectional	Study to establish the seroprevalence of infection by the dengue virus in a district of the Paraguayan Chaco
Pessanha, J.E.M., et al	2010	1	1	1		1	0	1			5	Cross-sectional	Study to determine dengue seroprevalence for to different viral serotypes in three districts in Belo Horizonte, Brazil Belo Horizonte
Piedrahita, L. D., et al.	2018	1	1	1		1	0	1			5	Cross-sectional	This longitudinal serological survey and spatial analysis study estimated dengue virus (DENV) transmission in schoolchildren (aged 5–19 years) in Medellin from 2010 to 2012
Rueda, J. C., et al	2019	1	1	1		0	0	1			4	Cross-sectional	The objective of this study was to describe the demographics and clinical characteristics of suspected chikungunya cases in six Colombian cities
Sissoko, D., et al.	2008	1	1	1		1	1	1			6	Cross sectional	Household-based cross sectional serosurvey to investigate the association between CHIKV seropositivity and risk factors
Soghaier, M. A., et al.	2015	1	1	1		1	1	1			6	Cross-sectional	The objective of this study was to identify socio-demographic factors associated with the prevalence of dengue serotypes in Kassala State in the eastern part of Sudan in 2011

Supplementary Table 1. Bias assessment using the Newcastle-Ottawa scale (NOS) for individual-level studies for cross-sectional and cohort (A) and case-control (B)

Author(s)	Year of publication	NOS Total	Study design	Study description
Bonifay, T., et al.	2017	6	Case (CHIKV)-control (DENV)	Study to describe the socioeconomic indicators of individuals infected with CHIKV and compare to those infected with DENV and the local population.
Luo, D., et al.	1995	8	Case control	Study examines children with Japanese encephalitis and compares them with neighborhood controls matched by age and sex in terms of several social and environmental variables.
Swain, S., et al.	2019	8	Case-control	The study aims to identify the social and ecological factors associated with emerging dengue in Odisha, India.
Udayanga, L., et al.	2018	4	Case-control	Evaluation of demographic, socio-economic an dother assoicated risk factors affecting the
