# Supplementary material for: Socioeconomic risk markers of arthropod-borne virus (arbovirus) infections: a systematic literature review and meta-analysis
Source: BMJ Glob Health. 2022 Apr 14;7(4):e007735. doi: 10.1136/bmjgh-2021-007735 (PMC9014035; doi:10.1136/bmjgh-2021-007735)
Supplement: Supplementary data [file bmjgh-2021-007735supp004.pdf]

Author(s)	Year	NOS	Country	Region	Study design	Unit of analysis (Individual or population)	Study description	Study year(s)	Study population: Source population	Study population: Total size	Study population: Sex	Study population: Age	Diagnosis/source of data	SINV: Test	SINV: Frequency measure	SINV: Incidence/Prevalence	CHIKV: Test	CHIKV: Frequency measure	CHIKV: Incidence/Prevalence	DENV: Test	DENV: Frequency measure	DENV: Incidence/Prevalence	JEV: Test	JEV: Frequency measure	JEV: Incidence/Prevalence	ZIKV: Test	ZIKV: Frequency measure	ZIKV: Incidence/Prevalence	WNV: Test	WNV: Frequency measure	WNV: Incidence/Prevalence	Any flavivirus or arbovirus: Test	Any flavivirus or arbovirus: Frequency measure	Any flavivirus or arbovirus: Incidence/Prevalence	RVFV: Test	RVFV: Frequency measure	RVFV: Incidence/Prevalence
Abd-Jamil, J. et al	2020		Malaysia	Orang Asli	Cross-sectional	Individual	To determine these factors by investigating the potential influence that demographic and socioeconomic variables as well as land cover and physical environmental factors might have on dengue IgG seroprevalence	2007-2010	Orange Asli populations residing in eight different OA villages in the forest or forest fringe areas of Peninsular Malaysia	491			Laboratory data							ELISA	Prevalence	17%															
Ahlm, C., et al.	2014		Sweden	Northern Sweden	Cross-sectional	Individual	Seroprevalence study to determine seroprevalence of SINV and associated risk factors.	2009	Random selection from population registers	1729	-	25-74	Clinical data	EIA for IgG																							
Al-Raddadi	2019		Saudi Arabia	Makkah, Madinah, Jeddah, Jizan	Cross-sectional	individual	To estimate the seroprevalence of dengue in these regions and the risk factors associated with positive serology	2017	Residents of the four cities of all genders, age groups, and SEC	6397	60.4% male, 39.6% female	8.2% from 0-10, 44.5% from 11-30, 47.2% > 30.	laboratory data							IgG ELISA	Prevalence	26.70%															
Anjos, R. et al	2020		Brazil	Salvador	Cross-sectional	individual	Investigate factors associated with prior CHIKV infection	2016-2017	All households of 3 contiguous valleys in Pau da Lima who are >=5 years of age and have slepted >= 3 nights per week in the house	1772	57% female		laboratory data				IgG/IgM ELISA + PRNT	prevalence	11.80%																		
Bartley, H., et al.	2002		Vietnam	Southern vietnam	Cross-sectional	Individual and household	Study assesses associations between sociodemograpgic factors and dengue and Japanese encflavivirus seroprevalence in Southern Vietnam.	Apr 1996- Aug 1997	Community and hospital-based subjects recruited for other sutdy (thypoid c-c study)	308			Clinical data							DENV/JEV IgG+	Prevalence	66% (95% CI 60.6 - 71.2)	JEV/DENV IgG+	Prevalence													
Bonifay, T., et al.	2017		French Guiana		Case (CHIKV)-control (DENV)	Individual	Study to describe the socioeconomic indicators of individuals infected with CHIKV and compare to those infected with DENV and the local population.	CHIKV (Apr-Jun 2014) DENV (Mar-Aug 2013)	Patients who sought treatment at hospital for CHIKV; Retrospective collection of control group of patients with acute DENV	168 and 168	-	>15yrs and 3months	Clinical diagnosis and laboratory confirmation				CHIKV RT-PCR+, DENV NS1-and IgM -			DENV IgM+																	
Brunkard, J. M., et al.	2007		USA	Texas/Mexico border	Cross-sectional	Household level	Cross-sectional serosurvey used to assess dengue seroprevalenceon the southern Texas-Mexico border and assess assocaited risk factors	Oct-Nov 2004		600 (300 households)			Laboratory							DENV IgM+ (DENV IgG+)	Prevalence	2-7.3% (40-78%)															
Burger-Calderon, R., et al.	2018		Nicaragua	Managua	Cohort	Individual and household	Study assessing the prevalence of ZIKV and its social determinants in Nicaragua.	Aug-Oct 2016	Participants of Nicaraguan Padiatric Dengue Cohort Study (PDCS) and national surveillance	33 laboratory confirmed index cases and 109 household members	-		Clinical data													RT-PCR	Incidence	31%									
Chiaravalloti-Neto, F., et al.	2019		Brazil	São Paulo	Cross-sectional	Individual	Seroprevalence study to determine seroprevalence and incidence of DENV and identify if SES and demographic covariates are associated with seropositivity.	Oct 2015 - Mar 2016	Residents of Vila Toninho neighbourhood	1322		≥10	Clinical data							DENV IgG+		74.60%															
Conlan, J. V., et al.	2015		Laos	Northern Laos	Cross-sectional	Individual	Seroprevalence study to determine seroprevalence of flaviviruses (JEV and DENV) and associated risk factors.	Jan-Mar 2009	Random selection of 14 households per village and all household members over 6yrs age asked to participate	1136		≥6	Clinical data							DENV	Prevalence	DENV1 (26.1,24.1,24.9,46.3%); DENV2 (26.1,24.1,24.9,46.3%); DENV3 (26.1,24.1,24.9,46.3%); DENV4 (26.1,24.1,24.9,46.3%)	JEV HI	Prevalence	JEV (54.6%) - of which 19.1% JEV only							DENV and JEV [illegible]	Prevalence	67.3 (64.6-[illegible])			

Author(s)	Exposure: Risk factors/Independent variables/Indicators	Categories	Frequency	Basic statistical analysis	Basic statistical analysis	Dependent variable	Statistical analysis	Crude effect measure type	Crude effect measure	Crude p	Adj Effect measure (95% CI)	Adj p	Effect	Confounders/Adjusted for
Abd-Jamil, J. et al	Sex	Female	57 (20.2%)			DENV seropositivity	Logistic regression	OR	1.78 (1.08-2.95)	0.023			Positive	Backwards elimination
		Male	26 (12.4%)						1					
	Age	>= 13	45 (34.6%)						4.43 (2.71-7.26)	<0.001	4.4		Positive	
		<=12	38 (10.6%)						1		1			
	Education	No formal education	41 (33.3%)						2.92 (2.00-4.27)	<0.001			Positive	
		Formal education	42 (11.4%)						1					
	Occupation status	Working	21 (33.9%)						2.34 (1.54-3.56)	<0.001			Positive	
		Not working	62 (14.5%)						1					
	Income	< RM 500	68 (20.7%)						2.23 (1.32-3.78)	<0.001	2.2		Positive	
		> RM 500	15 (9.3%)						1		1			
Ahlm, C., et al.	Sex	Males	3.7%	Chi2 or Student's t test	p=0.047	SINV seropositivity	Logistic regression analysis							Age, sex, smoking
		Females	2.0%											
	Age				p=0.14									
	Size of residential area	>15,000	1.3%		p≤0.001			OR			1			
		100-15000	4.4%								3.4 (1.6-7.3)	p=0.002	Positive	
		<1000	5.8%								4.3 (2.0-9.2)	<0.001	Positive	
	Level of education	No university studies	3.5%		p=0.013			OR			1			
		University studies	1.3%								0.42 (0.17-1.0)	0.054		
Al-Raddadi	Age group	0-10	60 (11.7%)			DENV seropositivity	Chi2	OR	1	p < 0.001	1			Multivariate analysis
		11-30	636 (22.3%)						2.16 (1.63-2.87)		2.10 (1.49-2.96)	p<0.001	Positive	
		>30	1014 (33.5%)						3.79 (2.86-5.01)		3.91 (2.78-5.50)	<0.001	Positive	
	Sex	Male	1057 (27.5%)						1.10 (0.98-1.23)	0.095	-	-		
		Female	653 (25.6%)						1		-	-		
	Housing type	Villa w/o garden	278 (30.1%)						1.93 (1.62-2.31)	<0.001	1.30 (1-1.69)	0.046	Positive	
		villa with garden	29 (21.9%)						1.25 (0.82-1.92)		0.81 (0.47-1.39)	0.447		
		Apartment (n=217)	418 (18.2%)						1		1			
		Popular house	606 (33.0%)						2.21 (1.92-2.55)		1.84 (1.56-2.17)	<0.001	Positive	
		Social house	341 (32.1%)						2. 12 (1.80-2.51)		1.82 (1.49-2.21)	<0.001	Positive	
		Other	5 (29.4%)						1.87 (0.66-5.34)		1.99 (0.55-7.27)	0.296		
	Number of rooms	1-3	387 (32.4%)						1.37 (0.95-1.96)	0.066				
		4-6	254 (28.0%)						1.11 (0.77-1.60)					
		7-10	100 (27.8%)						1.09 (0.73-1.65)					
		>10	45 (26%)						1					
	Number of occupants by household	<5	700 (27.9%)						1.33 (1.12-1.58)	0.007	1			
		6-10	740 (26.8%)						1.26 (1.06-1.50)		0.86 (0.74-0.99)	0.042	Negative	
		11-20	218 (22.6%)						1		0.71 (0.58-0.88)	0.002	Negative	
		>20	52 (31.3%)						1.57 (1.09-2.25)		1.99 (0.55-7.27)	0.708		
	Water interruption	Regular	843 (25.4)						1	0.001				
		Irregular	717 (29.3)						1.22 (1.09-1.37)					
	Sanitation	Present	816 (24.9)						1	0.001				
		Absence	865 (29.3)						1.25 (1.12-1.40)					
Anjos, R. et al	Sex	Male	93 (12.2%)	Chi2	p = 0.6	CHIKV seropositivity	Poisson regression	PR			N/A			N/A
		Female	116 (11.5%)											
	Age group	5-14	41 (8.1%)		p = 0.35									
		15-39	104 (11.9%)											
		>=40	63 (14.9%)											
	Skin color	Non-white	199 (12.0%)		p = 0.39									
		White	10 (9.1%)											
	Household per capita income in US$/day	≤5.5	171 (12.0%)		p = 0.69									
		>5.5	37 (10.9%)											
	Education	Illiterate	14 (18.4%)		0.06				1.60 (0.99-2.60)					
		Literate	195 (11.5%)						1					
	Occupation	Yes	60 (9.9%)		0.08				1					
		No	148 (12.7%)						1.28 (0.97-1.68)					
	Type of residence construction	Plastered wall	154 (10.9%)		0.04				1					
		Unplastered wall	33 (15.6%)						1.47 (0.92-2.35)					
		Wood or other material	21 (19.8%)						1.86 (1.06-3.28)					
	Residents/household	1	13 (9.0%)		0.31									
		2-3	89 (13.2%)											
		4-5	60 (9.0%)											
		≥6	5 (13.5%)											
Bartley, H., et al.	Age					Flavivirus seropositivity	Logistic regression	OR						
	Sex	M (n=158)	97 (61.4)						3.15 (1.44-6.85)		3.58 (1.45-8.87)			
		F (n=150)	106 (70.7)						0.74 (0.33-1.64)		1.07 (0.41-2.8)			
	Place of residence	Cao Lanh City (Urban) (n=172)	126 (73.3)						0.48 (0.30-0.77)		0.50 (0.28-0.89)			
		Surrounding district (n=136)	77 (56.6)											
	Level of education	No Schooling (n=25)	18 (72)											
		Primary school (n=178)	116 (65.2)											
		High school/College (n=105)	69 (65.7)											
	Television ownership	Yes (n=138)	88 (63.8)											
		No (n=170)	115 (67.6)											
Bonifay, T., et al.	Sex	Male	77 (45.8%) vs 91 (54.2%)		0.13	CHIKV vs DENV		OR	0.72 (0.46-1.12)	p=0.13	N/A			
		Female												
	Age group	<18	6 (3.6%) vs 22 (13.1%)		0.002				0.25 (0.08-0.65)	p=0.002				
		>50	67 (39.9%) vs 35 (20.8%)		<0.001									
	Migration status	Born in France *	117 (69.6) vs 58 (34.5)		<0.001				4.35 (2.69-7.06)	p<0.001				
		Born abroad												
	Health insurance	Yes	25 (14.9) vs 14 (8.3)		0.06				1.92 (0.92-4.16)	p=0.06				
		No												
	Social status (CMU (Free universal health care)/State medical aid)	Not precarious	90 (53.6) vs 55 (32.7)		<0.001				2.37 (1.49-3.78)	p<0.001				
		Precarious ^												
	Neighbourhood	Wealthy	123 (82.0) vs 69 (44.0)		<0.001				5.81 (3.35-10.2)	p<0.001				
		Poor $												
	Precarious social status	None	25 (14.9) vs 14 (8.3)		0.06				1.92 (0.92-4.16)	P=0.06				
		State medical aid (AME)	11 (6.6) vs 6 (3.6)		0.21				1.89 (0.62-6.37)	p=0.21				
	No precarious social	Free universal health care (CMU)	54 (32.1) vs 35 (20.8)		0.02				1.80 (1.07-3.05	p=0.02				
		Social secuity only	46 (27.4) vs 18 (10.7)		0.001				3.14 (1.68-6.04)	p=0.001				
		Persons with long term illness	10 (6.0) vs 7 (4.2)		0.45				1.46 (0.49-4.62)	p=0.45				
		Complimentary health insurance	22 (13.1) vs 88 (52.4)		<0.001				0.14 (0.08-0.24)	p≤0.001				
Brunkard, J. M., et al.	Income (<=$100)					Recent DENV	Poisson regressoin				3.2 (95% CI 1.31–7.95, p = 0.01)		Positive	Adjusted for study design
						Past DENV					2.59 (95% CI 1.58–4.26, p = 0.000)		Positive	
	Street drainage					Recent DENV					0.69 (95% CI 0.29–1.65, p = 0.395)			
						Past DENV					0.57 (95% CI 0.37–0.87, p = 0.009)		Negative	
	Air-conditioning					Recent DENV					0.74 (95% CI 0.28–1.96, p = 0.543)			
						Past DENV					0.58 (95% CI 0.38–0.89, p = 0.014)		Negative	
	Intact screens					Recent DENV					0.98 (95% CI 0.41-2.32, p = 0.959)			
						Past DENV					1.35 (95% CI 0.93–1.95, p = 0.111)			
	No. of people/household					Recent DENV					0.97 (95% CI 0.80–1.17, p = 0.727)			
						Past DENV					1.06 (95% CI 0.95–1.19, p = 0.300)			
Burger-Calderon, R., et al.	Sex (Female)					Positive contacts of ZIKV index cases		RR	0.79 (0.50-1.24)		0.80 (95% CI 0.51, 1.26)			Adjusted for refrigerator ownership
	Age								0.70 (0.42, 1.16)		0.68 (95% CI 0.41, 1.13)			
	Contacts								1.61 (0.84, 3.09)		1.60 (95% CI 0.83, 3.06)			
	Owning refrigerator								1.15 (0.59, 2.25)		N/A			
	Onsite water storage								2.27 (1.24, 4.16)		2.32 (95% CI 1.26, 4.28)		Positive	
Chiaravalloti-Neto, F., et al.	Sex	M (n=521)	391 (75)	N/A	Not included in paper			Posterior mean fixed effects (OR)	1		1			Adjusted for socioeconomic and demographic covariates - presumably sex, age, maritial status, age (standardised) schooling, occupation, income, house type, home ownership, hours at home, number of residents
		F (n=801)	595 (74.3)						0.99 (0.76-1.29)		0.96 (0.73-1.27)			
	Age								1.39 (1.21-1.60)		1.43 (1.21-1.70)		Positive	
	Race	White (n=679)	482 (71.0)						1		1		Positibve	
		non-white (n=535)	381 (78.4)						1.36 (1.04-1.78)		1.42 (1.08-1.89)			
	Matital status	Not married (n=566)	417 (73.7)						1		1			
		Married (n=746)	561 (75.2)						1.16 (0.89-1.51)		0.93 (0.70-1.25)			
	Occupation	Inside (n=345)	277 (80.3)						1		1			
		Outside (n=932)	680 (73.0)						0.70 (0.51-0.96)		0.91 (0.63-1.31)			
	Schooling	<=7 (n=596)	459 (77.0)						1		1			
		>7 (n=676)							0.80 (0.61-1.03)		1.01 (0.76-1.35)			
	House type	Apartment (n=217)	142 (65.4)						1		1		Positive	
		House (n=1105)	844 (76.4)						2.04 (1.10-3.78)		2.02 (1.10-3.73)			
	Home ownership	Owner (n=868)	646 (74.4)						1		1			
		Non-owner (n=441)	332 (75.3)						0.91 (0.69-1.21)		0.97 (0.72-1.31)			
	Income	0 to 2 MS (n=135)	114 (84.4)						0.89 (0.78-1.01)		0.89 (0.78-1.01)			
		2 to 4 MS (n=881)	660 (74.9)											
		4 MS or more (n=103)	67 (65.0)											
	No. of residents	<=4 (n=1027)	759 (73.9)						1		1			
		>4 (n=289)	222 (76.8)						1.13 (0.82-1.55)		1.33 0.95-1.86)			
Conlan, J. V., et al.	Wealth status	Most poor	1.8 (0.0-3.6)											
		Very poor	1.7 (0.0-3.4)											
		Poor	3.6 (1.3-5.9)											
		Less poor	1.6 (0.0-3.2)											
		Least poor	2.1 (0.1-4.2)											
		Most poor	0.9 (0.0-2.2)											
		Very poor	0.4 (0.0-1.3)											
		Poor	0.4 (0.0-1.2)											
		Less poor	0.4 (0.0-1.2)											
		Least poor	2.1 (0.1-4.2)											
		Most poor	0.5 (0.0-1.3)											
		Very poor	0.4 (0.0-1.3)											
		Poor	0.8 (0.0-1.9)											
		Less poor	1.6 (0.0-3.2)											
		Least poor	0.5 (0.0-1.6)											
		Most poor	8.5 (4.9-12.3)											
		Very poor	12.9 (8.6-17.2)											
		Poor	16.9 (12.2-21.5)											
		Less poor	21.2 (16.1-26.4)											
		Least poor	5.9 (2.5-9.2)											
		Most poor	27.6 (21.7-33.5)											
		Very poor	37.3 (31.1-43.6)											
		Poor	48.2 (42.0-54.4)											
		Less poor	41.6 (35.4-47.8)											
		Least poor	41.5 (34.4-48.6)											
		Most poor	45.2 (38.7-51.8)											
		Very poor	59.2 (52.9-65.6)											
		Poor	79.5 (74.5-84.5)											

																					Less poor	80.0 (74.9-85.0)												
																					Least poor	70.1 (64.2-77.3)												
Da Silva-Nunes, M., et al.	2008	Brazil	Brazilian Amazonia	Cross-sectional	Individual and household	Seroprevalence study to determine seroporevalence of DENV in Amazonas region of Brazil and associated risk factors	2004-2006		405			Clinical data	DENV IgG+	Prevalence	18.30%					Age group	5-14	8.1%	Chi2 for linear trend	p=0.0004	DENV seropositivity	Logistic regression analysis		OR	1					Migration history and other covariates
																					15-30	17.7%							2.43 (1.04–5.76)					
																					31-60	25.8%							3.93 (1.75–9.02)					
																					>60	33.3%							5.65 (1.72–18.65)					
																				Sex	Male	24.5%	Chi2 or Fisher exact test	p=0.001					2.53 (1.42–4.51)		2.60 (1.55-4.43)	p=0.001	Positive	
																					Female	11.4%							1		-			
																				Wealth index (quartiles)	1 (Poorest)	30.1%	Chi2 for linear trend	p=0.0005					4.05 (1.74–9.63)		0.88 (0.83-0.95)	p=0.001	Negative	
																					2	17.1%							1.94 (0.80–4.82)					
																					3	17.5%							1.99 (0.81–4.93)					
																					4 (Least poor)	9.1%							1					
																				Land ownership	Yes	16.8%	Chi2 or Fisher exact test	p=0.033					1					
																					No	31.7%							2.31 (1.03–4.90)					
																				House walls	Wood	19.2%		p=0.032					Undefined					
																					Brick	0.0%							1					
																				Wall gaps	Yes	18.5%		p=0.014					Undefined					
																					No	0.0%							1					
Eldigail, M.H. et al	2020	Sudan	Kassala state	Cross-sectional	individual	To determine the seroprevalence of Dengue in Kassala state and the associated risk factors.	8/2017-5/2018	11 localities of Kassala state	600			Laboratory data	ELISA	Prevalence	11.42%					Age group	≥5 and ≤18	18 (5.87%)	Chi2	p=0.001	DENV seropositivity	Logistic regression		OR	1	0.001			Positive	Excluded confounders not specified
																					>18	62 (15.8%)							3.24 (1.81-5.77)					
																				Sex	Female	32 (10.6%)		p=0.281										
																					Male	48 (12.6%)												
																				Level of education	Illiterate	19 (10.7%)		p=0.897										
																					Primary	30 (12.9%)												
																					Secondary	14 (9.6%)												
																					University	11 (12%)												
																					Informal study	6 (11.3%)												
																				Income	Low (<2000 SDG)	56 (12.7%)		p=0.136					3.75 (1.57-8.93)	0.027			Positive	
																					Medium (between 2000 and [illegible]	17 (11.6%)												
																					High (>3000 SDG)	7 (6.1%)							1					
																				Employment	Unemployed	33 (11.1%)		p=0.809										
																					Employed	47 (11.7%)												
Fournet, F., et al.	2016	Burkina Faso	Ouagadougou	Cross-sectional	Population	Seroprevalence study to analyse flavivirus prevalence relative to the socioeconomic, demographic, health and environmental data concerning children, their family and household and the district.	May-Sept 2004	Children from Ouagadougou districts with different types and degrees of urbanization	3015		0-12	Clinical data	DENV IgM+, DENV IgG+	Prevalence	IgG (22.7%)	Flavivirus IgM and IgG ELISA	Prevalence	22.7% (96 CI 22.4-26.7)		Sex	Male	20.7 (22.4–26.7)	Chi2	0.015	Past (IgG) Flavivirus seroprevalence	Logistic regression analysis	UHBD	OR	1					
																											ULBD							
																											SHBD							
																											SLBD							
																					Female	24.4 (18.7–22.8)					UHBD		0.92 (0.64–1.30)	ns				
																											ULBD		1.19 (0.85–1.67)	ns				
																											SHBD		1.83 (1.15–2.90)	p<0.05				
																											SLBD		1.25 (0.90–1.74)	ns				
																				Age group	0-4	11.8 (9.8–13.7)		<0.001			UHBD		1				Positive	
																											ULBD							
																											SHBD							
																											SLBD							
																					4-10	25.9 (23.7–28.2)					UHBD		6.52 (3.85–11.01)	p < 0.001				
																											ULBD		2.64 (1.76–3.96)	p < 0.001				
																											SHBD		1.91 (1.10–3.31)	p < 0.001				
																											SLBD		1.64 (1.10–2.45)	p < 0.001				
																					10-12	36.4 (32.3–40.9)					UHBD		11.09 (5.97–20.58)	p < 0.001				
																											ULBD		5.52 (3.32–9.18)	p < 0.001				
																											SHBD		2.72 (1.45–5.11)	p < 0.001				
																											SLBD		2.71 (1.69–4.38)	p < 0.001				
																				Education	Yes	21.6 (19.5–23.7)		0.15			UHBD		1					
																											ULBD							
																											SHBD							
																											SLBD							
																					No	24.3 (22.0–26.6)					UHBD		1.39 (0.95–2.01)	ns				
																											ULBD		1.44 (1.01–2.05)	p<0.01				
																											SHBD		1.17 (0.71–1.92)	ns				
																											SLBD		1.23 (0.88–1.71)	ns				
																				Socioeconomic level	High	22.4 (20.5–24.4)		0.003			UHBD		1					
																											ULBD							
																											SHBD							
																											SLBD							
																					Medium	26.5 (23.2–29.8)					UHBD		0.64 (0.24–1.66)	ns				
																											ULBD		1.65 (0.49–5.53)	ns				
																											SHBD		1.83 (0.99–3.39)	p<0.05				
																											SLBD		2.01 (1.21–3.33)	p<0.01				
																					Low	18.2 (14.8–21.5)					UHBD		0.47 (0.19–1.18)	ns				
																											ULBD		1.03 (0.39–4.31)	ns				
																											SHBD		1.20 (0.65–2.21)	ns				
																											SLBD		1.50 (0.91–2.47)	ns				
																				Water supply	Tap	22.2 (19.0–25.5)		0.03			UHBD		1					
																											ULBD							
																											SHBD							
																											SLBD							
																					Pump	21.9 (20.2–23.7)					UHBD		0.55 (0.25–1.22)	ns				
																											ULBD		7.39 (0.93–58.07)	p<0.05				
																											SHBD		0.76 (0.45–1.32)	ns				
																											SLBD		0.84 (0.57–1.24)	ns				
																					Well	28.9 (23.6–34.2)					UHBD		0.84 (0.32–2.23)	ns				
																											ULBD		10.27 (1.28–82.41)	p< 0.01				
																											SHBD		1.34 (0.31–5.75)	ns				
																											SLBD		1.22 (0.52–2.83)	ns				
																				Waste management	Improper	24.1 (22.0–26.3)		0.05			UHBD		1					
																											ULBD							
																											SHBD							
																											SLBD							
																					Adequate	21.2 (19.1–23.2)					UHBD		1.56 (1.05–2.34)	p<0.05				
																											ULBD		1.17 (0.82–1.66)	ns				
																											SHBD		1.92 (1.11–3.34)	p<0.01				
																											SLBD		0.90 (0.62–1.30)	ns				
																				Housing appearance	Good	24.4 (20.1–23.6)		0.12			UHBD		1					
																											ULBD							
																											SHBD							
																											SLBD							
																					Not good	21.9 (21.7–27.2)					UHBD		0.79 (0.49–1.29)	ns				
																											ULBD		2.16 (1.53–3.04)	p<0.001				
																											SHBD		0.56 (0.45–1.45)	ns				
																											SLBD		1.02 (0.68–1.53)	ns				
																				Strata	UHBD	20.2 (17.6–22.9)		<0.001										
																					ULBD	25.0 (22.1–28.0)												
																					SHBD	16.8 (13.6–19.7)												
																					SLBD	27.8 (24.4–30.9)												
																				Buidling density	High	18.9 (16.8–20.8)		<0.001										
																					Low	26.3 (24.1–28.5)												
Hortion, J., et al.	2019	Kenya	Western and coastal	Cohort	Individual	This seroprevalence study aimed to investigate the frequency of alphavirus and flavivirus incident infections in two regions in Kenya and identify potential risk factors	Dec 2014-Dec 2015	Acutely ill children presenting at one of four healthcare centres	1604							ELISA IgG CHIKV and IgG DENV	Prevalence	3.7% (60/1604)		Region	Western	2.3% (11/486)	Chi2 or Fisher exact test	p=0.044				OR						
																					Coastal	4.4% (49/1,118)												
																				Area type	Urban	3.6% (29/817)		p=0.70										
																					Rural	3.9% (31/787)												
																				Number of other children living in the hours (in addition to 1)	0													
																					1-2													
																					3-5													
																					6+													
																				Age	Young school children								19.2 (5.7-65.1)					
																					Older school children													
Jing, Q et al	2019	China	Guangzhou	Cross-sectional	Individual	Seroprevalence study to understand the prevalence of DENV in communities with no reported cases in Guangzhou, China and assess risk factors that may be associated with increased seropositivity.	2015	850 participants from 7 selected communities in Guangzhou with no reported dengue cases before 2014	850	F: 457 M: 393	between 1-84 yo	Laboratory data	IgG ELISA	Prevalence	6.59%					Age group	<14	(10) 3.86%	t-test		DENV seropositivity			OR	1		1.04 (1.01-1.07)	p = 0.02	Positive	
																					15-29	(6) 4.58%		0.42					1.54 (0.54-4.42)					
																					30-34	(13) 8.72%		0.03					2.53 (1.08-5.96)					
																					45-59	(13) 7.22%		0.09					2.07 (0.89-4.83)					
																					>=60	(14) 10.69%		0.01					2.84 (1.23-6.58)					
																				Gender	Female	(25) 5.47%		0.14					1					
																					Male	(31) 7.89%							1.52 (0.88-2.62)					
																				Level of education	Middle school and lower	(46) 6.61%		0.68					1.53 (0.20-11.87)					
																					Highschool	(9) 7.5%		0.48					2.11 (0.26-17.35)					
																					College degree and higher	(1) 2.94							1					
																				Housing Type	Multistory with elevator	(1) 3.03%							1					
																					Multistory without elevator	(41) 6.28%		0.46					2.21 (0.29-16.57)					
																					Single story	(14) 8.54%		0.45					3.08 (0.39-24.25)					
Kenneson, A., et al.	2017	Ecuador	Machala	Cross-sectional	Household level	The authors conducted a household-level study to identify KAP and social-ecological risk factors associated with acute or recent DENV infections in the city of Machala, Ecuador.	2014-2015	Individuals with DENV infections from sentinel clinics - as well as members of the same household and four neighboruing households located within 200m	219 households in total : 44 index HHs, 175 associate HHs			Clinical data	NS1 RDT, RT-PCR and IgM ELISA			DENV	Prevalence		Household with DENV	Age - mean (SD)		46.3 (12.8) vs 50.3 (14.7)	Chi2	0.04				OR						
																				Sex	Female	19.4% (27/139) vs 32.5% [illegible]		0.03										
																				Level of education	Secondary or higher	59.1% (81/137) vs 60.3% [illegible]		0.87										
																				Employment	If employed, make more than [illegible]	44.2% (50/113) vs 46.2% [illegible]		0.82										
																					If employed, employment is [illegible]	72.4% (89/123) vs 72.9% [illegible]		0.94										
																				Household characteristics	No. of people per HH - mean (SD)	4.73 (1.91) vs 4.29 (1.86)		0.1										
																					No. of bedrooms - mean (SD)	2.55 (1.04) vs 2.49 (1.10)		0.67										
																					No. of people per bedroom - [illegible]	2.18 (1.32) vs 2.02 (1.32)		0.39										
																					Rented property	19.4% (27/139) vs 13.8% [illegible]		0.29										
																					Adjacent abandoned property	37.0% (51/138) vs 22.8% [illegible]		0.03							2.89 (1.13-7.34)	p=0.03		
																					Screens on windows/doors	10.8% (15/139) vs 8.75% [illegible]		0.75										
																				Water supply	Piped water inside house	70.5% (98/139) vs 85.0% [illegible]		0.02							0.15 (0.05-0.47)	p=0.001		
																					Standing water	42.8% (59/138) vs 44.3% [illegible]		0.82										
																					Water containers covered	42.4% (59/139) vs 30.0% [illegible]		0.07										
Khan, J., et al.	2018	Pakistan	Swat	Cross-sectional		DENV patient samples	2013-2015		59765			Clinical data from hospital	RT-PCR	Incidence						Age group	1-13	9.1% (474/5190)	Chi2	<0.001										
																					14-30	52.5% (2727/5190)												
																					31-60	34.5% (1792/5190)												
																					Above 60	3.8% (197/5190)												
																				Level of education	Primary	38.4% (2115/5513)		<0.001										
																					Middle	29.5% (1627/5513)												
																					Bachelor	20.7% (1139/5513)												
																					Higher	11.5% (632/5513)												
																				Monthly income in Pakistani rupees	5000-10000	63.0% (3475/5513)		<0.001										
																					10001-20000	21.2% (3475/5513)												
																					20001-35000	10.4% (574/5513)												
																					Above 35000	5.4% (296/5513)												
																				Age (in years)														

Kikuti, M., et al.	2015	Brazil	Salvador	Cross-sectional	Individual and population	Conducted enhanced, community-based surveillance in the only public emergency unit in a slum in Salvador, Brazil to identify acute febrile illness (AFI) patients with laboratory evidence of dengue infection.	2009-2010		2962	>5	Clinical data	DENV IgM+. RT_PCR	Incidence		Self reported race	Black	49% (278/569)					OR	1.07 (1.05-0.90)		1.02 (1.01-1.04)			
																Mixed	39% (224/569)											
																White	9% (50/569)											
																Other	3% (17/569)											
															Illiterate partcipants		1% (5/352)					OR	1.13 (1.08-1.18)					
															Households with per							OR	1.04 (1.03-1.05)		1.02 (1.01-1.03)			
															Households with							OR	1.00 (0.99-1.01)					
															Households without public							OR	0.98 (0.92-1.04)					
															Households without							OR	1.02 (1.00-1.04)					
Kuan, G., et al.	2016	Nicaragua	Managua	Community based cohort	Individual	Two studies were conducted to analyse the seroprevalence of CHIKV after the first chikungunya epidemic in a community-based cohort of children ages 2-14 years and a cross-sectional survey of persons over 15 years old in the same are of Managua, Nicaragua.	Mar/Apr 2015 - 2016	Children aged 2–14 years enrolled in the Pediatric Dengue Cohort Study	3362	2-14yrs		ELISA	Prevalence	6.1% (95%CI 5.3-6.9)	Sex	Male	6.4 (5.3, 7.7)			CHIKV seropositivity		PR	1.13 (0.96-1.32)	ns	1.13 (0.97-1.33)[b]	ns		
																Female	5.7 (4.6, 6.9)						1		1			
															Age	2-4	4.8 (3.4, 6.5)						1		1			
																5-9	4.2 (3.2, 5.4)						0.87 (0.68-1.10)	ns	0.88 (0.69-1.11)[b]	ns		
																10-14	8.9 (7.4, 10.7)						1.86 (1.50-2.30)	p<0.05	1.85 (1.50-2.29)[b]	p<0.05	Positive	
															Socioeconomic status [a]	Not poor	4.8 (3.8, 6.1)						1		1			
																Poor	6.9 (5.9, 8.1)						1.42 (1.21-1.69)		1.33 (1.11-1.58)[b]	p<0.05	Positive	
															Daily hours without water	0	5.8 (4.9, 6.8)						1		1			
																1-7	6.2 (4.2, 8.7)						1.07 (0.85, 1.34)	ns	1.07 (0.85-1.34)[b]	ns		
																>8	7.7 (5.4, 10.5)						1.33 (1.06-1.65)	p<0.05	1.31 (1.05-1.63)[b]	p<0.05	Positive	
															Per additional person in								0.97 (0.95-0.99)	p<0.05	0.96 (0.95-0.98)[c]	p<0.05	Negative	
				Cross-sectional	Individual		Jun-15	individuals aged 15 years recruited door-to-door specifically for this study.	848	>15		ELISA	Prevalence	13.1% (95%CI 10.9-15.5)	Sex	Male	14.1 (9.9, 19.2)			CHIKV seropositivity		PR	1.11 (0.82-1.49)	ns	1.05 (0.78-1.41)	ns		
																Female	12.7 (10.2, 15.6)						1		1			
															Age	15-29	13.8 (9,7, 18.8)						1		1			
																30-44	10.3 (6.7, 15.2)						0.74 (0.50-1.10)	ns	0.70 (0.47-1.04)	ns		
																45-59	14.4 (9.8, 19.9)						1.04 (0.72-1.50)	ns	0.98 (0.68-1.41)	ns		
																>60	14.1 (9.5, 19.8)						1.02 (0.70-1.48)	ns	0.93 (0.64-1.36)	ns		
															Socioeconomic status [a]	Not poor	11.1 (7.9, 14.9)						1		1		Positive	
																Poor	14.4 (11.5, 17.8)						1.29 (0.97-1.72)	ns	1.11 (0.82-1.51)	ns		
															Daily hours without water	0	12.1 (9.7, 14.9)						1		1		Positive	
																1-7	17.8 (9.8, 28.5)						1.47 (0.95-2.26)	ns	1.48 (0.96-2.28)	ns		
																>8	15.2 (9.7, 22.3)						1.25 (0.88-1.78)	ns	1.25 (0.88-1.78)	ns		
															Per additional person in	-	-						0.97 (0.95-0.99)	p<0.05	0.96 (0.95-0.98)	p<0.05	Negative	
Liu, J., et al.	2018	China	Guangzhou	Cross-sectional		This cross-sectional study explored the sero-prevalence of dengue virus infection in Guangzhou	2013-2015	34 communities; All samples were selected from a 200,000-sample database holding serum collected from community residents living in Liwan and Yuexiu districts of Guangzhou between September 2013 and August 2015, and 17 to 28 individuals of each age group were chosen per month.	2085		Clinical data	IgG	Prevalence	11.80 (10.41–13.18)	Sex	Male	12.96 (10.57–15.34)	Chi2	0.212	Past CHIKV infection	Logistic regression	OR	1		-			None mentioned
																Female	11.13 (9.43–12.83)						0.84 (0.64–1.10)					
															Age	<19	1.68 (0.44–2.92)		0.000				1					
																19-40	5.08 (3.00–7.16)						3.13 (1.32–7.40)				Positive	
																41-65	7.07 (5.14 –9.00)						4.45 (1.99 –9.92)				Positive	
																>65	30.34 (26.51 –34.17)						25.45 (11.80 –54.89)				Positive	
															Level of education	Illiterate	14.47 (8.94–19.99)		0.000				1					
																Primary	16.25 (12.78–19.72)						1.15 (0.69–1.91)					
																Junior high school	13.85 (10.56–17.14)						0.95 (0.56–1.60)					
																Senior high school	8.25 (5.82–10.68)						0.53 (0.31–0.92)				Negative	
																Diploma and higher	8.1 (4.67–11.52)						0.52 (0.28–0.98)				Negative	
															Marital Status	Single	2.62 (1.26–3.98)		0.000				1					
																Married	13.94 (12.03–15.84)						6.01 (3.46–10.47)				Positive	
																Widowed	29.75 (21.49–38.02)						15.73 (8.14–30.39)				Positive	
																Divorced	16.67 (5.84–39.22)						7.43 (1.93–28.61)				Positive	
											IgM	Prevalence	3.98 (3.14–4.82)	Sex	Male	3.66 (2.33–5.00)	Chi2	0.575	Recent CHIKV infection	Logistic regression	OR	1					
																Female	4.16 (3.08–5.24)						1.14 (0.72–1.82)					
															Age	<19	4.33 (2.36–6.30)		0.399				1					
																19-40	3.23 (1.56–4.91)						0.73 (0.36 –1.51)					
																41-65	4.86 (3.24 –6.48)						?					
																>65	3.23 (1.76 –4.70)						0.74 (0.38 –1.44)					
															Educational status	Illerate	3.77 (0.78–6.77)		0.081				1					
																Primary	4.12 (2.25–5.99)						1.1 (0.43–2.81)					
																Junior high school	3.52 (1.76–5.28)						0.93 (0.35–2.44)					
																Senior high school	6.04 (3.94–8.14)						1.64 (0.67–4.01)					
																Diploma and higher	3.24 (1.02–5.46)						0.85 (0.29–2.51)					
															Marital Status	Single	3.56 (1.98–5.13)		0.340				1					
																Married	4.42 (3.28–5.55)						1.3 (0.76–2.20)					
																Widowed	1.65 (0.45–5.83)						0.46 (0.10–1.98)					
																Divorced	5.56 (0.99–25.76)						1.59 (0.20–12.61)					
Luo, D., et al.	1995	China	Southern Henan Province	Case-control	Population	A population-based case-control study in Southern Henan Province, central China, which examines children with Japanese encephalitis (JE) and compares them with neighborhood controls matched by age and sex in terms of several social and environmental variables.	Jun-Sept 1991	Active case finding in hospitals in Gusi County, Henan, China	50 cases, 100 controls	>6months - 10 years	JEV diagnosed clinically and confirmed by laboratory testing	IgG (HI)	Prevalence	61.90%	Income [a]	< 50%				Acute JEV infection	Logistic regression for matched case-control study	OR	1		1			Vaccination
																50-99%							0.66 (0.26-1.64)	p=0.038 *	0.96 (0.36-2.54)	p=0.235 *		
																> 100%							0.25 (0.06-1.02)		0.37 (0.09-1.57)			
															Parental education	≤ 5							1		1			
																6-8							0.49 (0.16-1.48)	p=0.006 *	0.55 (0.14-2.03)	p=0.163 *		
																≥ 9							0.09 (0.009-0.76)		0.26 (0.26-2.51)			
															Quality of house	Poor							1		1			
																General							0.71 (0.28-1.80)	p=0.072*	0.85 (0.29-2.47)	p=0.26*		
																Good							0.34 (0.10-1.15)		0.45 (0.12-1.72)			
Nakkhara, P., et al.	2013	Thailand	Southern Thailand	Cross-sectional		Estiamtion of whether symptomatic and asymptomatic chikungunya cases share the same risk factors	2008	Residents aged 18 years or more from three villages	507	>18	Clinical data	IgG (HI)	Prevalence	0.619	Age	18-36	55.1% (65/118)					RR		0.003				
																37-45	63.6% (82/129)											
																46-58	64.1% (84/131)											
																59-91	64.3% (83/129)											
															Occupation	Rubber farmer	64.6% (256/396)							<0.001				
																Other	46.0% (23/50)											
																None	57.4% (35/61)											
															Level of education	None	66.0% (31/47)							0.047				
																Early primary school	67.0% (144/215)											
																Primary school	56.9% (70/123)											
																High school	58.2% (57/98)											
																Diploma or higher	52.9% (12/24)											
Nasir, I. A., et al.	2017	Nigeria	Abuja	Cross-sectional	Individual	A hospital-based cross-sectional study investigated the prevalence of Dengue virus non-structural protein-1 (NS1) antigenaemia, anti-Dengue virus IgG and their associated risk factors among febrile patients attending the University of Abuja Teaching Hospital (UATH), Nigeria.	May-Aug 2016	Patients with febrile illesses seeking medical assistance at hospital	171			DENV NS1 ELISA	Prevalence	8.80%	Sex	Male		Chi2	-	DENV NS1 seropositivity								
																Female												
															Residential area	Suburb			-									
																Village												
															Education	No formal			-									
																Primary												
																Secondary												
																Tertiary												
															Occupation	Civil servant	8 (15.1%)		0.166									
																Farmer	0 (0.0)											
																Housewife	4 (12.5)											
																Unemployed	1 (4.3)											
																Other	2 (4.2)											
											DENV IgG ELISA	Prevalence	43.30%	Sex	M	28 (38.9%)	Chi2	0.406	DENV IgG seropositivity								
																F	46 (46.5)											
															Residential area	Suburb	64 (41.8)		0.3896									
																Village	10 (55.6)											
															Level of education	No formal	29 (76.3)		<0.0001									
																Primary	12 (38.7)											
																Secondary	16 (44.4)											
																Tertiary	17 (25.8)											
															Occupation	Civil servant	10 (20.8)		0.0034									
																Farmer	10 (66.7)											
																Housewife	14 (50.0)											
																Unemployed	10 (43.5)											
																Other	30 (52.6)											
Obaidat, M. M. and A. A. Roess	2018	Jordan		Cross-sectional	Individual	Seroprevalence study to understand the prevalence of DENV in Jordan and assess risk factors that may be associated with increased seropositivity.		Healthy relatives of patients at governmental human health centres at 11 governorates	892			DENV 1-4 IgG antibodies SERION enzyme-linked immunosorbent assay classic dengue virus IgG	Prevalence	24.6% (95% CI 21.8-27.5)	Age	<20	17.4			DENV seropositivity			1	-	1	1		
																20-39	21.1						1.27	p= 0.40	1.22 (0.69-2.15)	1.23 (0.70-2.17)		
																40-59	27						1.75	p= 0.07	1.62 (0.92 - 2.85)	1.64 (0.93-2.90)		
																60-80	36.7						2.75	p= 0.00	2.29 (1.19 - 4.43)	2.34 (1.21-4.54)		
															Sex	F	24.2						1	-	1	1		
																M	24.9						1.04	p= 0.80	0.99 (0.71 - 1.37)	0.80 (0.56-1.15)		
															Residential area	Rural	20.51						1	-				
																Urban	25.16						1.3	p= 0.28				
															Education	No	29.6						1	-	1	1		
																Yes	22.8						0.07	p= 0.04	0.84 (0.57 - 1.24)	0.83 (0.56-1.22)		
															Rainwater collection		18.35						0.66	p= 0.11				
															Filtered water								1.19	p= 0.29				
															Municipal water								0.84	p= 0.34				
															Has garden								1.33	p= 0.08				
															Practices agriculture								1.4	p= 0.05	1.25 (0.87 - 1.79)	1.22 (0.85-1.76)		
Obaidat, M. M., et al.	2019	Jordan		Cross-sectional		Countrywide study of the seroprevalence, risk factors and spatial distribution of West Nile virus in Jordan.	Nov 2015- May 2016	Health relatives of patients at governmental human health centres at 11 governorates	801		Clinical data, health centres	anti-WNV IgG using an ELISA kit	Prevalence	8.61% (95% CI 6.8-10.8)	Sex	M	11			WNV seropositivity		OR	1.62 (0.99-2.66)	p= 0.055				
																F	7.1						1					
															Age (cont.)								1.03 (1.10-1.04)	p<0.001				
															Age group	6-15	15.4						4.09 (0.81-20.54)	p=0.09				
																15-29	4.3						1					
																30-49	9.5						2.37 (1.18-4.78)	p=0.02				
																≥50	12.6						3.23 (1.60-6.51)	p=0.00				
															Level of education	No Education	15.6						3.33 (1.60-6.93)	p=0.00				
																School	7						1.35 (0.61-2.99)	p=0.46				
																College	4.6						0.88 (0.31-2.48,)	p=0.81				
																University degree	5.3						1					
															Any education	No education	15.6						2.96 (1.79-4.88)	p=0.00				
																Any education	5.9						1					
															Household income	≤US$750	10.2						2.0 (1.11-3.85)	p=0.02				
																>US$750	5.2						1					
												IgG (ELISA and ELISPOT-MNT)	Prevalence	12.5% (95% CI 8.7–16.3)	Sex	M	12.3 (8.0–16.5)					OR	1					
																F	12.6 (7.9–17.4)						1.03 (0.65–1.64)	p=0.89				
															Age group	15-29	8.7 (4.1–13.2)						1					
																30-49	13.3 (9.3–17.4)						1.62 (0.84–3.12)	p=0.06	1.51 (0.74–3.10)	p=0.26		
																50-64	23.9 (9.4–38.3)						3.3 (1.22–8.9)		3.06 (0.99–9.52)	p=0.05		
															Level of education	No primary	25.8 (16.4–35.2)						3.44 (1.47–8.06)	p< .01	1.20 (0.43–3.38)	p=0.73		
																Incomplete primary	6.5 (3.8–9.2)						0.69 (0.34–1.4)		0.54 (0.22–1.35)	p=0.19		
																Complete primary + secondary	9.2 (4.5–13.8)						1					
															Wealth Quintiles	Lowest	18.4 (11.2–25.7)						1.87 (0.82–4.25)	p=0.04	0.50 (0.20–1.26)	p=0.14		
																2nd	11.6 (4.0–19.2)						1.08 (0.41–2.89)		0.46 (0.16–1.34)	p=0.16		
																Middle	9.4 (4.9–14.0)						0.86 (0.36–2.06)		0.44 (0.16–1.22)	p=0.11		
																4th	11.5 (6.3–16.7)						1.07 (0.48–2.41)		0.72 (0.24–2.15)	p=0.56		
																Highest	10.8 (4.2–17.4)						1					
															Sex	M	5.4 (2.4–8.5)						1					
																F	3.7 (2.2–5.3)						0.67 (0.40–1.15)	p=0.15				
																15-29	3.3 (1.6–5.1)						1					

Ochieng, C., et al.	2015	Kenya		Cross-sectional	Individual	Seroprevalence study to understand the prevalence of DENV, CHIKC and RVFC in Kenya and associated risk factors	2007	HIV-negative blood specimens from the 2007 Kenya AIDS Indicator Survey	1091		15-64	Clinical/laboratory	IgG ELISA	Prevalence	4.5% (95% CI 2.7–6.3)		Age	30-49	4.2 (1.1–7.4)			DENV seropositivity		OR	1.28 (0.57–2.86)	p=0.05	0.83 (0.32–2.11)	p=0.69		Geography
																		50-64	8.7 (3.1–14.2)						2.76 (1.22–6.26)		2.25 (0.90–5.62)	p=0.08		
																	Education level	No primary	10.0 (3.5–16.4)						4.69 (2.05–10.72)		0.92 (0.48–1.79)	p=0.81		
																		Incomplete primary	3.4 (0.3–6.4)						1.48 (0.52–4.20)	p<0.01	0.97 (0.31–3.05)	p=0.96		
																		Complete primary + secondary	2.3 (1.1–3.5)						1					
																	Wealth Quintiles	Lowest	11.1 (5.3–16.9)						7.81 (2.25–27.13)		4.22 (0.72–24.64)	p=0.11		
																		2nd	4.4 (0.0–8.9)						2.87 (0.61–13.42)	p=<0.01	1.68 (0.23–12.34)	p=0.61		
																		Middle	2.7 (0.4–5.1)						1.77 (0.44–7.11)		1.18 (0.19–7.22)	p=0.85		
																		4th	2.2 (0.1–4.3)						1.40 (0.32–6.06)		0.93 (0.12–6.89)	p=0.94		
																		Highest	1.6 (0.0–3.3)						1					
													IgG ELISA	Prevalence	1% (95% CI 0.2–1.7)		Sex	Male	0.1 (0.0–0.4)					OR	1		12.09 (1.46–99.86)	p=0.02		
																		Female	1.5 (0.3–2.6)						11.54 (1.40–95.38)	p=0.02				
																	Age group	15-29	1.5 (0.3–2.6)						3.37 (0.80–14.15)	p=0.15	3.57 (0.84–15.21)	p=0.09		
																		30-49	0.4 (0.0–1.1)						1					
																		50-64	-											
																	Education level	No primary & incomplete primary	1.6 (0.2–3.1)					OR	4.38 (0.75–25.49)	p=0.1				
																		Complete primary + secondary	0.4 (0.0–0.9)						1					
																	Wealth Quintiles	Lowest	0.7 (0.0–1.9)						1.87 (0.20–17.13)					
																		2nd	1.3 (0.0–3.1)						3.37 (0.40–28.07)	p=0.47				
																		Middle	0.9 (0.0–2.3)						2.41 (0.31–18.96)					
																		4th	2.3 (0.0–5.5)						5.98 (0.77–46.60)					
																		Highest	0.4 (0.0–0.9)						1					
Omatola, C.A. et al	2020	Nigeria	Guinea Savannah	Cross-sectional	Individual	Assess seroprevalence of DENV in Guinea Savannah and highlighting associated risk factors	2019	Visiting outpatients from the four hospitals in Anyigba	200	39% females, 61% males		Laboratory data	IgG immunoassay	Prevalence	20.50%		Sex	Male	18 (23.1%)	Chi-square	p = 0.66			None						None.
																		Female	25 (20.5%)											
																	Age	<15	6 (21.4%)		p = 0.45									
																		15-29	12 (15.6%)											
																		30-34	15 (24.6%)											
																		45-59	7 (35.0%)											
																		60-74	3 (25.0%)											
																		>74	0											
																	Level of education	None	13 (27.1%)		p = 0.46									
																		Primary	6 (19.4%)											
																		Secondary	15 (24.2%)											
																		Tertiary	9 (15.3%)											
																	Occupation	Farming	7 (24.1%)		p=0.09									
																		Civil servant	5 (17.9%)											
																		Business	19 (31.7%)											
																		Student	12 (14.5%)											
																	Marital status	Married	26 (28.3%)		p = 0.12									
																		Divorced	0											
																		Widowed/Separated	3 (23.1%)											
																		Single	14 (15.7%)											
																	fomatola	1	13 (16.3%)		p = 0.14									
																		>=2	30 (25.0%)											
Omatola, C.A. et al	2020	Nigeria	Kogi state	Cross-sectional	individual	To determine seroprevalence of active and past CHIKV infection among febrile patients who test negative for malaria and typhoid fever and determine assoicated risk factors.	2018	Febrile participants at five hospitals in Anyigba who test negative for typhoid and malaria	243			Laboratory data	IgG and IgM detection kit	Prevalence	34.20%		Sex	Male	45 (40.9%)	Chi-squared	p = 0.044	CHIKV seropositivity	Logistic regression	OR	1.7 (1.014-2.955)				Positive	None mentioned
																		Female	38 (28.6%)						1					
																	Age	5-14	2 (5.4%)		p < 0.001								Positive	
																		15-29	17 (22.7%)											
																		30-34	41 (49.4%)											
																		45-59	11 (42.3%)											
																		60-74	9 (56.3%)											
																		75-80	3 (50.0%)											
																	Level of education	None	13 (32.5%)		p = 0.43									
																		Primary	7 (21.9%)											
																		Secondary	30 (36.6%)											
																		Tertiary	33 (37.1%)											
																	Occupation	Farming	25 (48.1%)		p<0.001									
																		Civil servant	15 (38.5%)											
																		Business	26 (44.1%)											
																		Student	17 (18.3%)											
																	Marital status	Married	51 (45.5%)		p = 0.002								Positive	
																		Divorced	7 (43.8%)											
																		Widowed	6 (27.3%)											
																		Single	19 (20.4)											
Pereira, Y., et al	2015	Paraguay	Paraguayan Chaco	Cross-sectional	Individual	Study to establish the seroprevalence of infection by the dengue virus in a district of the Paraguayan Chaco	2014	Inhabitants of three villages	418			Household sampling confirmed by laboratory testing	DENV IgG ELISA	Prevalence	24.2% (95% CI 20.2% to 28.6%)		Sex	Male	31.7% (45/142)		0.009	DENV seropositivity								
																		Female	20.3% (56/276)											
																	Age group	<10	16.7% (1/6)		<0.001									
																		10-19	11.9% (5/42)											
																		20-40	13% (24/185)											
																		41-60	34.5% (38/110)											
																		>=60	44% (33/71)											
																	Level of education	Illiterate	33.3% (6/18)		0.366									
																		Complete primary	27.3% (56/205)											
																		Complete secondary	20.4% (29/142)											
																		Tertiary / university	41.2% (10/53)											
																	Occupation	Housewife	18.1% (28/155)		0.003									
																		Merchant	29.5% (21/71)											
																		Labourer	39.7% (25/63)											
																		Student	9.3% (4/43)											
																		University professional	31.4% (11/35)											
																		Public official	22.6% (7/31)											
																		Domestic worker	15.4% (3/13											
																		Unemployed	42.9% (3/7)											
																	Monthly income	Less than minimum wage	24.1% (28/116)		0.736									
																		More than the minimum wage	25.3% (56/221)											
																		Has no income	21% (17/81)											
																	Live alone	Yes	42.4% (14/33)		0.008									
																		No	22.6% (87/385)											
																	Housing type	Plastic/mud	0		0.099									
																		Wood	11.5% (6/52)											
																		Wall/brick	26.2% (95/363)											
																	Water service for consumption	Tap water	23.3% (83/373)		0.332									
																		Bottled water	27.7% (13/47)											
																		Water well	33.3% (5/15)											
Pessanha, J.E.M., et al	2010	Brazil	Belo Horizonte	Cross-sectional	Individual	Study to determine dengue seroprevalence for to different viral serotypes in three districts in Belo Horizonte, Brazil Belo Horizonte	June 2006 and March 2007	All residents aged over 1 year in the threee Belo Horizonte districts (Venda Nova, DS Leste and DS Centro-Sul)	709			Questionnaire and blood samples	Serology to different DENV serotypes (type and lab test not specified)	Prevalence	11.9% (96% CI 9.7% to 14.6%)		Sex	Female	11.24% (48/427)			DENV seropositivity		OR	1					
																		Male	12.77% (36/284)						1.14 (0.70-1.85)	0.58				
																	Age group	<15 or >60 years	8.81% (20/227)						1					
																		15 to 60 years	13.28% (64/482)						1.58 (0.93-2.69)	0.09				
																	Family income	> 2 * minimum wage	19.90% (54/417)						1					
																		Up to 2 * minimum wage	11.60% (75/632)						0.80 (0.46-1.38)	0.39				
																	Health vulnerability index	Low	8.14% (14/172)						1					
																		Average	12.10% (34/281)						1.56 (0.81-2.99)	0.19				
																		High	14.70% (29/197)						1.95 (0.99-3.82)	0.05				
																		Very high	11.90% (7/59)						1.52 (0.58-3.97)	0.39				
																	Type of housing	Apartment	5.30% (5/77)						1					
																		House/shack	12.80% (79/615)						2.63 (1.03-8.53)	0.02				
																	Changed city	Yes	11.69% (9/77)						1					
																		No	11.87% (75/632)						1.02 (0.47-2.28)	0.96				
Piedrahita, L. D., et al.	2018	Colombia	Medellin	Longitudinal serosurvey	Population and individual	This longitudinal serological survey and spatial analysis study estimated dengue virus (DENV) transmission in schoolchildren (aged 5–19 years) in Medellin from 2010 to 2012.	2010-2012	School children	4385		5-19yrs	Schoolchildren	IgG (ELISA and ELISPOT-MNT)	Incidence	53.8%(2010) to 64.6%(2012)	2010	Sex	Male	52.9% (459/867)			DENV IgG seropositivity		OR	1		1			
																		Female	54.6% (503/921)						1.03 (0.95-1.12)		1.03 (0.84-1.25)	p=0.77		
																	Ethnicity	Afro-descent	45.6% (41/90)						1					
																		Mestizo	54.2% (921/1,698)						1.19 (0.95-1.50)					
																	Age group	5-9	34.9% (199/571)						1		1			
																		10-14	61.6% (594/965)						2.99 (2.41-3.71)	p < 0.05	2.96 (2.38-3.68)	p<0.001		
																		15-19	67.1% (169/252)						3.80 (2.78-5.21)	p < 0.05	3.71 (2.70-5.09)	<0.001		
																	Socioeconomic status	Low	58.2% (592/1,017						1		1			
																		Middle	49.1% (318/648)						0.67 (0.57-0.84)	p < 0.05	0.70 (0.57-0.86)	p=0.001		
																		High	42.3% (52/123)						0.53 (0.36-0.77)	p < 0.05	0.59 (0.39-0.87)	p=0.009		
																2011	Sex	Male	57.2% (510/891)						1					
																		Feamle	59.6% (594/997)						1.04 (0.96-1.12)					
																	Ethnicity	Afro-descent	63.1% (41/65)						1					
																		Mestizo	58.8% (1,058/1,800)						0.93 (0.77-1.13)					
																	Age	5-9	45.4% (181/399)						1		1			
																		10-14	60.2% (660/1,097)						1.90 (1.51-2.30)	p < 0.05	1.90 (1.51-2.40)	p<0.001		
																		15-19	67.1% (264/392)						2.60 (1.95-3.47)	p < 0.05	2.59 (1.94-3.46)	p<0.001		
																	Socioeconomic status	Low	60% (682/1,137)						1		1			
																		Middle	56.5% (368/651)						0.87 (0.72-1.6)		0.86 (0.71-1.05)	p=0.150		
																		High	54.6% (53/97)						0.80 (0.53-1.22)		0.91 (0.59-1.39)	p=0.663		
																2012	Gender	M	59.9% (200/334)						1					
																		F	68.8% (258/375						1.15 (1.03-1.28)		1.38 (1.10-1.89)	p=0.043		
																	Ethnicity	Afro-descent	71.4% (10/14)						1					
																		Mestizo	64.5% (448/695)						0.90 (0.64-1.26)					
																	Age	5-9	50% (94/188)						1		1			
																		10-14	70.5% (292/414)						2.40 (1.68-3.41)	p < 0.05	2.32 (1.62-3.31)	<0.001		
																		15-19	64.6% (72/107)						2.06 (1.25-3.40)	p < 0.05	1.97 (1.20-3.24)	0.008		
																	Socioeconomic status	Low	61.2% (210/343)						1					
																		Middle	68% (247/363)						1.37 (1.0-1.85)	p < 0.05				
																		High	0											
Rueda, J.C. et al	2019	Colombia		Cross-section nested in community cohort	Individual	Describe the demographic and clinical characteristics of CHIKV cases	2014	548 suspected CHIKV patients from the COPCORD cohort	548	F: 382 M: 166	>18 yo	Laboratory data	IgM or IgG ELISA	Prevalence	53.80%		Age group	>45 yo	(170) 57.6%	t-test		CHIKV seropositivity		OR						None mentioned
																		<45 yo	(125) 42.4%											
																	Sex	Female	(208) 73.3%											
																		Male	(87) 52.4%											
																	Monthy income	None	(107) 53.8%						2.13 (1.32-3.46)	0.002			Positive	
																		<157 USD	(62) 68.9%											
																		158-315 USD	(95) 50.3%											
																		316-471 USD	(16) 39.0%											
																		472-630 USD	(9) 60.0%											
																		631-725 USD	(2) 66.7%											
																		726-906 USD	(3) 42.9%											
																		>906 USD	(1) 25.0%											
																	Socioeconomic strata (higher number = more economic capacity)	Stratum 1	(115) 68.0%						2.35 (1.60-3.44)	<0.001			Positive	
																		Stratum 2	(104) 46.0%						0.58 (0.41-0.82)	0.002			Negative	
																		Stratum 3	(60) 52.6%											
																		Stratum 4	(9) 28.1%						0.31 (0.14-0.69)	0.03			Negative	
																		Stratum 5	(6) 85.7%						-					
																		Mestizo	145 (49.7%)											

																		Race	Caucasian	110 (58.5%)											
																			Afro-American	29 (70.7%)											
																			Amerindian	8 (33.3%)											
																		Education (not in table-- in text only)	at least Primary school education	-						1	p = 0.048			Positive	
																			No education	-						1.43 (1-2.05)					
Sissoko, D., et al.	2008	Mayotte (France)		Cross sectional	Individual	Household-based cross sectional serosurvey to investigate the association between CHIKV seropositivity and risk factors	2005-2006	Household-based; complex multistage cluster sampling of population of Mayotte	1154	Clinical data	IgG ELISA	Prevalence	37.2% (95% CI= 33.9-40.5)					Sex	Male	40.6 (32.6–48.7)	Adjusted Wald X2 test	p=0.03			OR						
																			Female	33.8 (23.8–43.9)											
																		Age group	2-14	33.2 (24.2–42.2)		p=0.33									
																			15-24	39.3 (27.2–51.5)											
																			25-34	41.7 (29.7–53.7)											
																			35-44	40.2 (29.7–50.8)											
																			45-54	26.9 (15.1–38.8)											
																			>55	36.6 (23.3–49.8)											
																		Length of education	0-6y	42.1 (32.3–51,9)		p=0.003									
																			>6y	25.7 (16.4–35.1)											
																		Occupation	Employed	28.6 (19.9–37.3)		p=0.035									
																			Schooled	34.2 (22.1–46.2)											
																			Unemployed/housewife	44.8 (33.5–56.2)											
																		Construction type	Concrete	30.2 (21.9–38.4)		p=0.058									
																			Adobe and stone	44.2 (31.1–57.4)											
																			Makeshift	65.6 (47.6–83.6)											
																		Household size	1-2	35.1 (24.4–45.8)		p=0.054									
																			3-4	46.7 (36.6–56.9)											
																			>5	34.3 (24.6–44.1)											
																		Asset index	Below median threshold	61.2 (45.9–76.5)		p=0.0009									
																			At or over median threshold	31.6 (23.8–39.4)											
Soghaier, M. A., et al.	2015	Sudan		Cross-sectional	Indiviudal and community	The objective of this study was to identify socio-demographic factors associated with the prevalence of dengue serotypes in Kassala State in the eastern part of Sudan in 2011.	2011	Randomly selected community population through multi-stage cluster sampling	540	Clinical data				IgG ELISA	Prevalence	9.4 % (95 % CI: 7.1–12.3)		Age group	<35	10.0% (26/281)		p=0.69	DENV seropositivity		OR	1					
																			>35	8.7% (18/206)						1.17 (0.55-2.5)	p=0.69				
																		Sex	Male	10.1% (29/288)		0.24				1.55 (0.74-3.25)	p=0.24				
																			Female	8.5% (17/199)						1					
																		Level of education	No formal education	11.1% (16/144)		0.67				0.84 (0.38-1.86)	p=0.67				
																			Formal education	8.7% (30/344)						1					
																		Water source	Not piped water	12.8% (6/47)		0.56				1.35 (0.49-3.72)	p=0.56				
																			Piped water	9.1% (40/438)						1					
																		Household density	>3	13.5% (24/178)		0.034				2.08 (1.06-4.09)	p=0.034				
																			<3	7.1% (22/311)						1					
Swain, S. et al	2020	India	Odisha	Case-control	individual	Identify the social and ecological factors associated with emerging dengue in Odisha, India	2017	Confirmed dengue patients within one year in six districts of the state	387 cases and 380 controls	national vector borne disease control program (tested through IgM)				ELISA				Sex	Male	0.525	none			logistic analysis	OR	Matched					Adjusted for other variables - not specified
																			Female	0.478											
																		Age group	<15	0.127						Matched					
																			16-25	0.233											
																			26-35	0.222											
																			36-45	0.186											
																			>=46	0.233											
																		Level of education	illterate	0.147						1		1			
																			Primary	0.191						1.2 (0.7-1.9)		0.8 (0.47-1.5)			
																			Secondary	0.424						1.2 (0.8-1.8)		0.9 (0.54-1.5)			
																			High school or above	0.238						2.5 (1.4-4.2)		1.4 (0.76-2.8)			
																		Ethnicity	Non SC/ST	0.155						1		1			
																			SC/ST	0.845						1.6 (1.1-2.3)		1.4 (0.9-2.1)			
																		Occupation	Unemployed	0.098						1		1			
																			Agriculture	0.194						3.9 (2.1-7.5)		3.8 (1.8-7.9)	p < 0.05	Positive	
																			Business	0.088						3.5 (1.7-7.4)		4.6 (1.9-10.6)	p < 0.05	Positive	
																			Home maker/student	0.465						4.6 (2.6-8.1)		4.4 (2.3-8.4)	p < 0.05	Positive	
																			Industry/office	0.155						2.7 (1.4-5.2)		3.0 (1.4-6.5)	p < 0.05	Positive	
																		Housing	Asbestos	0.199						1		1			
																			Concrete	0.346						1.9 (1.3-2.9)		1.9 (1.2-3.1)	p < 0.05	Positive	
																			Mixed	17.8]%						1.6 (1.1-2.6)		2.0 (1.1-3.5)	p < 0.05	Positive	
																			Thatched	0.276						2.6 (1.7-4.1)		2.9 (1.7-5.1)	p < 0.05	Positive	
Udayanga, L., et al.	2018	Sri Lanka		Case-control	Household level	Evaluation of demographic, socio-economic an dother assoicated risk factors affecting the occurrence of DENV incidence.	Feb-Apr 2017	Random selection of 200 households reporting past dengue incidence and 200 non-dengue reported households	2000 cases, 2000 controls								Kandy	Age (Control, case n(%)	0-15	15 (1.50) vs 355 (35.5)	Chi2	p=0.04									
																			15-35	277 (27.7) vs 422 (42.2)											
																			35-55	495 (49.5) vs 155 (15.5)											
																			>55	213 (21.3) vs 66 (6.6)											
																		Sex	M	903 (90.30) vs 608 (60.8)		p=0.01									
																			F	970 (9.70) vs 392 (39.2)											
																		Family size	1-3	165 (16.5) vs 316 (31.6)		p=0.04									
																			4-6	520 (52.0) vs 504 (50.4)											
																			>7	315 (31.5) vs 180 (18.0)											
																		Monthly income	<5	3 (0.3) vs 4 (0.4)		p=0.02									
																			5-10	20 (2.0) vs 46 (4.6))											
																			10-20	55 (5.5) vs 258 (25.8)											
																			23-30	605 (60.5) vs 472 (47.2)											
																			>30	317 (31.7) vs 220 (22.0)											
																	Colombo	Age	0-15	18 (1.8) vs 207 (20.7)		p=0.03									
																			15-35	302 (30.2) vs 488 (48.8)											
																			35-55	517 (51.7) vs 230 (23.0)											
																			>55	162 (16.2) vs 75 (7.5)											
																		Sex	M	883 (88.3) vs 586 (58.6)		p=0.03									
																			F	117 (11.7) vs 414 (41.4)											
																		Family size	1-3	247 (24.7) vs 396 (39.6)		p=0.03									
																			4-6	458 (45.8) vs 498 (49.8)											
																			>7	295 (29.5) vs 106 (10.6)											
																		Monthly income	<5	0 (0) vs 5 (0.5)		p=0.008									
																			5-10	0 (0) vs 35 (3.5)											
																			10-20	50 (5.0) vs 395 (39.5)											
																			23-30	266 (26.6) vs 275 (27.5)											
																			>30	684 (68.4) vs 290 (29.0)											
