# Supplementary material for: Socioeconomic risk markers of arthropod-borne virus (arbovirus) infections: a systematic literature review and meta-analysis
Source: BMJ Glob Health. 2022 Apr 14;7(4):e007735. doi: 10.1136/bmjgh-2021-007735 (PMC9014035; doi:10.1136/bmjgh-2021-007735)
Supplement: Abstract translation [file bmjgh-2021-007735supp005.pdf]

Resumo

Introdução

Os arbovírus possuem notável importância em saúde pública em todo o mundo devido ao seu potencial de causar grandes surtos e de gerar manifestações clínicas debilitantes ou possivelmente fatais. Esta revisão sistemática e meta-análise tem como objetivo avaliar a relação entre indicadores de posição socioeconômica (SEP, sigla em inglês) e infecção por arboviroses com mosquitos vetores.

Métodos

Realizamos uma busca sistemática nas bases de dados Pubmed, Embase e LILACS para identificar estudos publicados entre 1980 e 2020 medindo a associação entre marcadores de SEP e infecção por arboviroses. Incluímos os estudos observacionais, sem realizar restrições por localização geográfica ou idade. Excluímos estudos da literatura cinzenta, revisões e ecológicos. Os dados dos estudos foram extraídos, resumidos e utilizada a meta-análise com efeitos aleatórios para obtenção das estimativas combinadas de efeito.

Resultados

Identificamos 36 estudos observacionais usando dados referentes a 106.524 participantes do estudo em 23 localizações geográficas que examinaram empiricamente a relação entre indicadores socioeconômicos e infecções causadas por sete arbovírus (vírus da Dengue, Chikungunya, Encefalite Japonesa, Febre do Vale do Rift, Sindbis, Febre do Nilo Ocidental e Zika). Embora os resultados tenham variado, a síntese

descritiva apontou um maior risco de infecção por arboviroses associado a indicadores de SEP mais baixos, incluindo menor escolaridade, menor renda, baixa cobertura de saúde, materiais de habitação precários, abastecimento de água interrompido, estado civil (casado, solteiro, divorciado ou viúvo), etnias não brancas e status migratório. As estimativas brutas combinadas indicaram um risco aumentado de infecção por arboviroses associado à baixa escolaridade (RR = 1,5 IC 95%: 1,3, 1,9); I2=83,1%), interrupção do abastecimento de água (RR = 1,2; IC 95%: 1,1,1,3; I2 = 0,0%) e ser casado anteriormente (RR = 1,5 IC 95%: 1,1, 2,1; I2=85,2%).

Conclusão

As evidências dessa revisão sistemática sugerem que pior SEP aumenta o risco de adquirir infecção por arbovírus. No entanto, houve grande heterogeneidade entre os estudos. Mais estudos são necessários para definir a relação entre indicadores específicos de SEP a nível individual, domiciliar e comunitário e a infecção por arbovírus para informar intervenções direcionadas de saúde pública.

Palavras-chave: posição socioeconômica, equidade em saúde, infecção por arbovírus, mosquitos vetores, revisão sistemática
