# Supplementary material for: Socioeconomic risk markers of arthropod-borne virus (arbovirus) infections: a systematic literature review and meta-analysis
Source: BMJ Glob Health. 2022 Apr 14;7(4):e007735. doi: 10.1136/bmjgh-2021-007735 (PMC9014035; doi:10.1136/bmjgh-2021-007735)
Supplement: Abstract translation [file bmjgh-2021-007735supp006.pdf]

Resumen

Introducción

Los arbovirus son de notable importancia para la salud pública global por su potencial de causar brotes explosivos además de manifestaciones clínicas debilitantes y potencialmente letales. Esta revisión sistemática y meta-análisis tiene como objetivo la evaluación de la relación entre indicadores de posición socioeconómica (PS) e infecciones por arbovirus transmitidos por mosquitos vectores.

Métodos

Realizamos una búsqueda sistemática en las bases de datos Pubmed, Embase, y LILACS para identificar estudios publicados entre 1980 y 2020 que median la asociación entre marcadores de PS e infección arboviral. Incluimos estudios observacionales sin restricciones sobre la localidad geográfica o edad de los participantes. Excluimos estudios de la literatura gris, revisiones y estudios ecológicos. Los hallazgos de los estudios fueron extraídos y resumidos y se realizaron meta-análisis de efectos aleatorios para obtener estimaciones combinadas de efecto.

Resultados

Identificamos 36 estudios observacionales con datos pertenecientes a 106,524 participantes de 23 localidades geográficas que examinaron empíricamente la relación entre factores socioeconómicos e infecciones causadas por siete arbovirus (Dengue, Chikungunya, Encefalitis Japonesa, Fiebre del Valle de Rift, Sindbis, Fiebre del Nilo Occidental, y el Zika). Mientras que los resultados fueron variados, la síntesis descriptiva señaló un riesgo mayor de

infección arboviral asociada con marcadores de PS más bajos, incluyendo menor nivel educativo, escasez de ingresos, baja cobertura de saneamiento, materiales de viviendas de baja calidad, interrupciones del suministro de agua, estado civil (casado, soltero, divorciado o viudo), grupos étnicos no caucásicos y estatus migratorio. Las estimaciones agrupadas brutas indicaron un riesgo aumentado de infección arboviral asociado con menor nivel educativo (RR = 1.5 95% CI: 1.3, 1.9); I^2=83.1%), interrupciones del suministro de agua (RR = 1.2; 95% CI: 1.1,1.3; I^2 = 0.0%) y haber estado casado (RR = 1.5 95% CI: 1.1, 2.1; I^2=85.2%).

Conclusión

Esta revisión sistemática señala que el tener una PS inferior aumenta el riesgo de adquirir infección arboviral, sin embargo hubo una gran heterogeneidad entre los estudios. Más estudios son necesarios para mejor definir la relación entre indicadores de PS individuales, a nivel de hogar, y a nivel comunitario y la infección arboviral para mejor diseñar intervenciones de salud pública dirigidas.
